# Multi-Modal Latent Diffusion

**DOI:** 10.3390/e26040320

**Published:** 2024-04-05

**Authors:** Mustapha Bounoua, Giulio Franzese, Pietro Michiardi

**Affiliations:** 1 Ampere Software Technology, 06560 Valbonne, France; 2 Department of Data Science, EURECOM, 06410 Biot, France; giulio.franzese@eurecom.fr (G.F.); pietro.michiardi@eurecom.fr (P.M.)

**Keywords:** multimodality, generative models, score-based models, diffusion models

## Abstract

Multimodal datasets are ubiquitous in modern applications, and multimodal Variational Autoencoders are a popular family of models that aim to learn a joint representation of different modalities. However, existing approaches suffer from a coherence–quality tradeoff in which models with good generation quality lack generative coherence across modalities and vice versa. In this paper, we discuss the limitations underlying the unsatisfactory performance of existing methods in order to motivate the need for a different approach. We propose a novel method that uses a set of independently trained and unimodal deterministic autoencoders. Individual latent variables are concatenated into a common latent space, which is then fed to a masked diffusion model to enable generative modeling. We introduce a new multi-time training method to learn the conditional score network for multimodal diffusion. Our methodology substantially outperforms competitors in both generation quality and coherence, as shown through an extensive experimental campaign.

## 1. Introduction

Multi-modal generative modeling is a crucial area of research in machine learning that aims to develop models capable of generating data according to multiple modalities, such as images, text, audio, and more. This is important because real-world observations are often captured in various forms; thus, combining multiple modalities describing the same information can be an invaluable asset. For instance, images and text can provide complementary information in describing an object, while audio and video can capture different aspects of a scene. Multimodal generative models can help in tasks such as data augmentation [1,2,3], missing modality imputation [4,5,6,7], and conditional generation [8,9].

Multimodal models have flourished over the past years and seen tremendous interest from academia and industry, especially in the content creation sector. Whereas most recent approaches focus on specialization, by considering text as a primary input to be associated mainly with images [10,11,12,13,14,15,16] and videos [17,18,19], in this work we target an established literature with more general scope and in which all modalities are considered equally important.

Multi modal generative models aim at *high-quality* data generation, as well as at generative *coherence* across all modalities. These objectives apply to both joint generation of new data and to conditional generation of missing modalities given a disjoint set of available modalities. The predominant literature in this field is based on extensions of the Variational Autoencoder (VAE) [20] to the multimodal domain; initially interested in learning joint latent representation of multimodal data, such works have mostly focused on generative modeling.

In short, multimodal VAEs relies on combinations of unimodal VAEs, and the design space mainly consists of the way in which the unimodal latent variables are combined to construct the joint posterior distribution. Early works such as [21] adopted a product-of-experts approach, whereas others [22] considered a mixture-of-experts approach. While product-based models achieve high generative quality, they suffer in terms of both joint and conditional coherence. This has been found to be due to mis-calibration issues on the part of the experts [22,23]. On the other hand, mixture-based models produce coherent but qualitatively poor samples. A first attempt to address the so-called **coherence–quality tradeoff** [24] was represented by the mixture of products of experts approach [23]. However, recent comparative studies [24] have shown that none of the existing approaches fulfill the criteria of both generative quality and coherence. A variety of techniques are aimed at finding a better operating point, such as contrastive learning techniques [25], hierarchical schemes [26], total correlation-based calibration of single-modality encoders [27], and different training objectives [28]. More recently, in [29], explicitly separated shared and private latent spaces were considered as a way to overcome the aforementioned limitations.

In Section 2, we investigate the limitations of multimodal VAEs and prepared the ground to substantiate a new approach which overcomes the shortcomings in the state of the art. We further investigate the tradeoff [24] between generative coherence and quality, and argue that it is intrinsic to all variants of multimodal VAEs. We indicate two root causes of the problem: latent variable collapse [30,31] and information loss due to mixture subsampling. To tackle these issues, in Section 3 of this work we propose a new approach that uses a set of independent and unimodal *deterministic* autoencoders with the latent variables simply concatenated in a joint latent variable. Joint and conditional generative capabilities are provided by an additional model that learns a probability density associated with the joint latent variable. We propose an extension of score-based diffusion models [32] to operate on the multimodal latent space. Thus, we derive both forward and backward dynamics that are compatible with the multimodal nature of the latent data. In Section 4, we propose a novel multi-time diffusion process that can both be used for joint and conditional generation. We label our approach Multi-modal Latent Diffusion (MLD).

Our experimental evaluation of MLD in Section 5 provides compelling evidence of the superiority of our approach for multimodal generative modeling. We compare MLD to a large variety of VAE-based alternatives on several real-life multimodal datasets in terms of generative quality and both joint and conditional coherence. Our model outperforms alternatives in all possible scenarios, even those that are notoriously difficult because the modalities might be only loosely correlated. We note that recent works have explored the joint generation of multiple modalities [33,34]; however, such approaches are application-specific, e.g., text-to-image, and essentially only target two modalities. When relevant, we compare our method to additional recent alternatives to multimodal diffusion [35,36] and show the superior performance of MLD.

## 2. Limitations of Multimodal VAEs

In this work, we consider multimodal VAEs [21,22,23,29] as the standard modeling approach to tackle both joint and conditional generation of multiple modalities. Our goal here is the need to go beyond such a standard approach in order to overcome limitations that affect multimodal VAEs, which result in a tradeoff between generation quality and generative coherence [24,29].

Consider the random variable $X=\left\{X^{1},\ldots ,X^{M}\right\}∼p_{D}\left(x^{1},\ldots ,x^{M}\right)$, consisting of the set *M* of modalities sampled from the (unknown) multimodal data distribution $p_{D}$. We indicate the marginal distribution of a single modality by $X^{i}∼p_{D}^{i}\left(x^{i}\right)$ and the collection of a generic subset of modalities by $X^{A}∼p_{D}^{A}\left(x^{A}\right)$, with $X^{A}\overset{\mathrm{def}}{=}{\left\{X^{i}\right\}}_{i\in A}$, where A⊂{1,…,M} is a set of indexes; for example, given A={1,3,5}, we would have $X^{A}=\left\{X^{1},X^{3},X^{5}\right\}$.

We begin by considering unimodal VAEs as particular instances of the Markov chain $X\to Z\to \hat{X}$, where *Z* is a latent variable and $\hat{X}$ is the generated variable. Models are specified by the two conditional distributions, called the encoder $Z\;{|}_{X=x}∼q_{\psi }\left(z\;|\;x\right)$ and decoder $\hat{X}\;{|}_{Z=z}∼p_{\theta }\left(\hat{x}\;|\;z\right)$. For a given prior distribution $p_{n}\left(z\right)$, the objective is to define a generative model with samples that are distributed as similarly as possible to the original data.

In the case of multimodal VAEs, we consider the general family of Mixture of Product of Experts (MOPOE) [23], which includes as particular cases many existing variants such as Product of Experts (MVAE) [21] and Mixture of Expert (MMVAE) [22]. Formally, a collection of *K* arbitrary subsets of modalities $S=\{A_{1},\ldots A_{K}\}$ along with weighting coefficients $\omega_{i}\geq 0,\sum_{i=1}^{K}\omega_{i}=1$ define the posterior $q_{\psi }\left(z\;|\;x\right)=\sum_{i}\omega_{i}q_{\psi^{A_{i}}}^{i}\left(z\;|\;x^{A_{i}}\right)$, with $\psi =\{\psi^{1},\ldots ,\psi^{K}\}$. To lighten the notation, we use $q_{\psi^{A_{i}}}$ in place of $q_{\psi^{A_{i}}}^{i}$, noting that the various $q_{\psi^{A_{i}}}^{i}$ can have both different parameters $\psi^{A_{i}}$ and functional forms. For example, in the MOPOE [23] parametrization, we have $q_{\psi^{A_{i}}}\left(z\;|\;x^{A_{i}}\right)=\prod_{j\in A_{i}}q_{\psi^{j}}\left(z\;|\;x^{j}\right)$. Our exposition is more general, and is not limited to this assumption. The selection of the posterior can be understood as the result induced by the two step procedure where (i) each subset of modalities $A_{i}$ is encoded into specific latent variables $Y_{i}∼q_{\psi^{A_{i}}}\left(\cdot \;|\;x^{A_{i}}\right)$ and (ii) the latent variable *Z* is obtained as $Z=Y_{i}$ with probability $\omega_{i}$. Optimization is performed with respect to the following evidence lower bound (ELBO) [23,24]:

(1) $$L=\sum_{i}\omega_{i}\int p_{D}\left(x\right)q_{\psi^{A_{i}}}\left(z\;|\;x^{A_{i}}\right)\log p_{\theta }\left(x|z\right)-\log\frac{q_{\psi^{A_{i}}}\left(z\;|\;x^{A_{i}}\right)}{p_{n}\left(z\right)}dzdx.$$

A well known limitation called the latent collapse problem [30,31] affects the quality of the latent variables *Z*. Consider the hypothetical case of arbitrary flexible encoders and decoders. Posteriors with zero mutual information with respect to model inputs are valid maximizers of Equation (1). To prove this, it is sufficient to substitute the posteriors $q_{\psi^{A_{i}}}\left(z\;|\;x^{A_{i}}\right)=p_{n}\left(z\right)$ and $p_{\theta }\left(x|z\right)=p_{D}\left(x\right)$ into Equation (1) to observe that the optimal value of $L=\int p_{D}\left(x\right)\log p_{D}\left(x\right)dx$ is achieved [30,31]. The problem of information loss is exacerbated in the case of multimodal VAEs [24]. Intuitively, even if the encoders $q_{\psi^{A_{i}}}\left(z\;|\;x^{A_{i}}\right)$ carry relevant information about their inputs $X^{A_{i}}$, step (ii) of the multimodal encoding procedure described above induces a further information bottleneck. Some fraction $\omega_{i}$ of the time, the latent variable *Z* will be a copy of $Y_{i}$, which only provides information about the subset $X^{A_{i}}$. No matter how good the encoding step is, the information about $X^{\{1,\ldots ,M\}\setminus A}$ that is not contained in $X^{A_{i}}$ cannot be retrieved.

The variable collapse problem can be analyzed through the lenses of self-reconstruction, whereby a multimodal VAE is evaluated by simply reconstructing the same modality it receives as input. We have observed that these models tend to encode input samples into a latent space with possible information loss, leading to inconsistent reconstruction. This is particularly shown by the quantitative results in Table A7, with notable difficulty in reconstructing the SVHN modality.

Furthermore, if the latent variable carries zero mutual information with respect to the multimodal input, a coherent *conditional* generation of a set of modalities given others is impossible, as ${\hat{X}}^{A_{1}}⊥X^{A_{2}}$ for any generic sets $A_{1},A_{2}$. While the factorization $p_{\theta }\left(x\;|\;z\right)=\prod_{i=1}^{M}p_{\theta^{i}}\left(x^{i}\;|\;z\right)$, $\theta =\{\theta_{1},\ldots ,\theta_{M}\}$ (we use $p_{\theta^{i}}$ here instead of $p_{\theta^{i}}^{i}$ to unclutter the notation) could enforce preservation of information and guarantee better quality of the *jointly* generated data, in practice the latent collapse phenomenon induces multimodal VAEs to converge towards suboptimal a operating regime. When the posterior $q_{\psi }\left(z\;|\;x\right)$ collapses onto the uninformative prior $p_{n}\left(z\right)$, the ELBO in Equation (1) reduces to the sum of modality-independent reconstruction terms:

(2) $$\begin{array}{c} \sum_{i}\omega_{i}\sum_{j\in A_{i}}\int p_{D}^{j}\left(x^{j}\right)p_{n}\left(z\right)\left(\log p_{\theta^{j}}\left(x^{j}|z\right)\right)dzdx^{j} \end{array}$$

where, paradoxically, the quality of the approximation of the various marginal distributions is extremely high, while there is a complete lack of joint coherence.

General principles to avoid latent collapse involve explicitly forcing the learning of informative encoders $q_{\theta }\left(z\;|\;x\right)$ via β− annealing of the Kullback-Leibler (KL) term in the ELBO and the reduction of the representational power of encoders and decoders. While β− annealing [37] has been explored in the multimodal VAEs literature, [21] with limited improvements reported, reducing the flexibility of the encoders/decoders clearly impacts the generation quality. Hence, the presence of the tradeoff; in order to improve coherence, the flexibility of encoders/decoders should be constrained, which in turn impacts generative quality. This tradeoff has recently been addressed in the literature on multimodal VAEs [24,29]; however, our experimental results in Section 5 indicate that there is ample room for improvement and that a new approach is truly needed.

## 3. Our Approach: Multimodal Latent Diffusion

**Figure 1:**
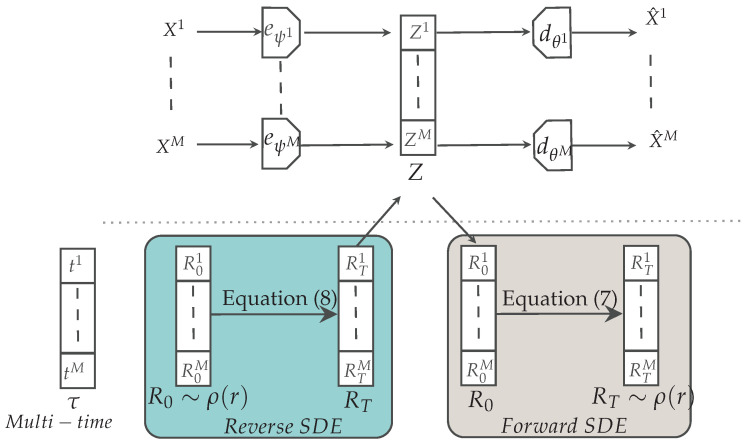
Multimodal Latent Diffusion: two-stage model involving (**Top**): deterministic modality-specific encoder/decoders and (**Bottom**): the score-based diffusion model on the latent spaces of the modalities, which evolve differently through the diffusion process according to a multi-time vector.

We propose a new method for multimodal generative modeling that by design does not suffer from the limitations discussed in Section 2. Our objective is to enable both high quality and coherent joint/conditional data generation using a simple design (see Figure 1 for a schematic representation). As an overview, we use deterministic unimodal autoencoders whereby each modality $X^{i}$ is encoded through its encoder $e_{\psi^{i}}$ (which is a short form for $e_{\psi^{i}}^{i}$) into the modality-specific latent variable $Z^{i}$ and decoded into the corresponding ${\hat{X}}^{i}=d_{\theta^{i}}\left(Z^{i}\right)$. Our approach can be interpreted as a latent variable model in which the different latent variables $Z^{i}$ are concatenated as $Z=[Z^{1},\ldots ,Z^{M}]$. This corresponds to the parameterization of the two conditional distributions as $q_{\psi }\left(z\;|\;x\right)=\prod_{i=1}^{M}\delta \left(z^{i}-e_{\psi^{i}}\left(x^{i}\right)\right)$ and $p_{\theta }\left(\hat{x}\;|\;z\right)=\prod_{i=1}^{M}\delta \left({\hat{x}}^{i}-d_{\theta^{i}}\left(z^{i}\right)\right)$, respectively. Then, in place of an ELBO, we optimize the parameters of our autoencoders by minimizing the following sum of modality-specific losses:

(3) $$L=\sum_{i=1}^{M}L_{i},\;L_{i}=\int p_{D}^{i}\left(x^{i}\right)l^{i}\left(x^{i}-d_{\theta^{i}}\left(e_{\psi^{i}}\left(x^{i}\right)\right)\right)dx^{i},$$

where $l^{i}$ can be any valid distance function, e.g, the square norm ‖·‖^2^. The parameters $\psi^{i},\theta^{i}$ are modality-specific; thus, minimization of Equation (3) corresponds to individual training of the different autoencoders. Because the mapping from input to latent is deterministic, there is no loss of information between *X* and *Z* (note that as the measures are not absolutely continuous with respect to the Lebesgue measure, the mutual information is +∞). Moreover, this choice avoids any form of interference in the backpropagated gradients corresponding to the unimodal reconstruction losses. Consequently, gradient conflict issues [38], in which stronger modalities pollute weaker ones, are avoided.

To enable such a simple design to become a generative model, it is sufficient to generate samples from the induced latent distribution $Z∼q_{\psi }\left(z\right)=\int p_{D}\left(x\right)q_{\psi }\left(z\;|\;x\right)dx$ and decode them as $\hat{X}=d_{\theta }\left(Z\right)=\left[d_{\theta^{1}}\left(Z^{1}\right),\ldots ,d_{\theta^{M}}\left(Z^{M}\right)\right]$.

To obtain such samples, we follow the two-stage procedure described in [39,40,41], where samples from the lower-dimensional $q_{\psi }\left(z\right)$ are obtained through a score-based generative model. These models have shown tremendous performance in fitting complex distributions [10,42], an ability which aligns with our objective of learning the distribution within a multimodal latent space. Furthermore, the conditioning mechanism inherent in score-based models facilitates highly coherent generation. MLD is further enhanced by a multi-time diffusion process, a novel mechanism that allows for the generation of any subset of modalities, and which we explain in Section 4.

It may be helpful at this point to clarify that the two-stage training of MLD is carried out separately. Unimodal deterministic autoencoders are pretrained first, followed by the training of the score-based diffusion model, which is explained in more detail later.

To conclude this overview of our method, for joint data generation it is possible to sample from noise, perform backward diffusion, and then decode the generated multimodal latent variable to obtain the corresponding data samples. For conditional data generation, given one modality, the reverse diffusion is guided by this modality, while the other modalities are generated by sampling from noise. The generated latent variable is then decoded to obtain data samples of the missing modality.

### Joint and Conditional Multimodal Latent Diffusion Processes

In the first stage of our method, the deterministic encoders project the input modalities $X^{i}$ into the corresponding latent spaces $Z^{i}$. This transformation induces a distribution $q_{\psi }\left(z\right)$ for the latent variable $Z=[Z^{1},\ldots ,Z^{M}]$, resulting from the concatenation of unimodal latent variables.

**Joint generation:** To generate a new sample for all modalities, we use a simple score-based diffusion model in latent space [32,39,40,42,43]. This requires reversing a stochastic noising process, starting from a simple Gaussian distribution. Formally, the noising process is defined by a Stochastic Differential Equation (SDE) of the form

(4) $$dR_{t}=\alpha \left(t\right)R_{t}dt+g\left(t\right)dW_{t},\;\;\;R_{0}∼q\left(r,0\right),$$

where $\alpha \left(t\right)R_{t}$ and g(t) are the drift and diffusion terms, respectively, and $W_{t}$ is a Wiener process. The time-varying probability density q(r,t) of the stochastic process at time t∈[0,T], where *T* is finite, satisfies the Fokker–Planck equation [44] with initial conditions q(r,0). We assume the uniqueness and existence of a stationary distribution ρ(r) for the process in Equation (4), though this is not necessary for the validity of the method [45]. The forward diffusion dynamics depend on the initial conditions $R_{0}∼q\left(r,0\right)$. We consider $R_{0}=Z$ to be the initial condition for the diffusion process, which is equivalent to $q\left(r,0\right)=q_{\psi }\left(r\right)$. Under loose conditions [46], a time-reversed stochastic process exists, with a new SDE of the form

(5) $$dR_{t}=\left(-\alpha \left(T-t\right)R_{t}+g^{2}\left(T-t\right)\nabla \log\left(q\left(R_{t},T-t\right)\right)\right)dt+g\left(T-t\right)dW_{t}\;\;\;R_{0}∼q\left(r,T\right),$$

indicating that, in principle, simulation of Equation (5) allows samples to be generated from the desired distribution q(r,0). In practice, we use a **parametric score network** $s_{\chi }\left(r,t\right)$ to approximate the true score function, and we approximate q(r,T) with the stationary distribution ρ(r). Indeed, the generated data distribution q(r,0) is close (in the KL sense) to the true density as described by [45,47]:

(6) $$\mathrm{KL}[q_{\psi }\left(r\right)\;|\;\;|\;q\left(r,0\right)]\leq \frac{1}{2}\int_{0}^{T}g^{2}\left(t\right)E\left[{\|s_{\chi }\left(R_{t},t\right)-\nabla \log q\left(R_{t},t)\right\|}^{2}\right]dt+KL[q\left(r,T\right)||\rho \left(r\right)]$$

where the first term on the right-hand side is referred to as the score-matching objective, and is the loss over which the score network is optimized, while the second is a vanishing term for T→∞.

To conclude, joint generation of all modalities is achieved through simulation of the reverse-time SDE in Equation (5), followed by a simple decoding procedure. Indeed, optimally trained decoders (achieving zero in Equation (3)) can be used to transform $Z∼q_{\psi }\left(z\right)$ into samples from $\int p_{\theta }\left(x\;|\;z\right)q_{\psi }\left(z\right)dz=p_{D}\left(x\right)$.

**Conditional generation.** Given a generic partition of all modalities into non-overlapping sets $A_{1}\cup A_{2}$, where $A_{2}=\left(\left\{1,\ldots ,M\right\}\setminus A_{1}\right)$, conditional generation requires samples from the conditional distribution $q_{\psi }\left(z^{A_{1}}\;|\;z^{A_{2}}\right)$, which are based on *masked* forward and backward diffusion processes.

Given conditioning latent modalities $z^{A_{2}}$, we consider a modified forward diffusion process with initial conditions $R_{0}=C\left(R_{0}^{A_{1}},R_{0}^{A_{2}}\right)$ and with $R_{0}^{A_{1}}∼q_{\psi }\left(r^{A_{1}}\;|\;z^{A_{2}}\right),R_{0}^{A_{2}}=z^{A_{2}}$. The composition operation C(·) concatenates generated ($R^{A_{1}}$) and conditioning latents ($z^{A_{2}}$). As an illustration, consider $A_{1}=\left\{1,3,5\right\}$ such that $X^{A_{1}}=\left\{X^{1},X^{3},X^{5}\right\}$ and $A_{2}=\left\{2,4,6\right\}$ such that $X^{A_{2}}=\left\{X^{2},X^{4},X^{6}\right\}$; then, $R_{0}=C\left(R_{0}^{A_{1}},R^{A_{2}}\right)=C\left(R_{0}^{A_{1}},z^{A_{2}}\right)=\left[R_{0}^{1},z^{2},R_{0}^{3},z^{4},R_{0}^{5},z^{6}\right]$.

More formally, we define the following masked forward-diffusion SDE:

(7) $$dR_{t}=m\left(A_{1}\right)⊙\left[\alpha \left(t\right)R_{t}dt+g\left(t\right)dW_{t}\right],\;\;\;q\left(r,0\right)=q_{\psi }\left(r^{A_{1}}\;|\;z^{A_{2}}\right)\delta \left(r^{A_{2}}-z^{A_{2}}\right)$$

The mask $m(A_{1})$ contains *M* vectors $u^{i}$, one per modality, with the corresponding cardinality. If modality $j\in A_{1}$, then $u^{j}=1$; otherwise, $u^{j}=0$. Then, the effect of masking is to “freeze” the part of the random variable $R_{t}$ corresponding to the conditioning latent modalities $z^{A_{2}}$ throughout the diffusion process. We naturally associate the conditional time-varying density $q\left(r,t\;|\;z^{A_{2}}\right)=q\left(r^{A_{1}},t\;|\;z^{A_{2}}\right)\delta \left(r^{A_{2}}-z^{A_{2}}\right)$ with this modified forward process.

To sample from $q_{\psi }\left(z^{A_{1}}\;|\;z^{A_{2}}\right)$, we derive the reverse-time dynamics of Equation (7) as follows:

(8) $$dR_{t}=m\left(A_{1}\right)⊙\left[\left(-\alpha \left(T-t\right)R_{t}+g^{2}\left(T-t\right)\nabla \log\left(q\left(R_{t},T-t\;|\;z^{A_{2}}\right)\right)\right)dt+g\left(T-t\right)dW_{t}\right]$$

with initial conditions $R_{0}=C\left(R_{0}^{A_{1}},z^{A_{2}}\right)$ and $R_{0}^{A_{1}}∼q\left(r^{A_{1}},T\;|\;z^{A_{2}}\right)$. Then, we approximate $q(r^{A_{1}},T\;|\;z^{A_{2}})$ by its corresponding steady-state distribution $\rho (r^{A_{1}})$ and the true (conditional) score function $\nabla \log(q\left(r,t\;|\;z^{A_{2}}\right))$ by a conditional score network $s_{\chi }\left(r^{A_{1}},t\;|\;z^{A_{2}}\right)$.

## 4. Multi-Time Diffusion to Learn the Conditional Score Network

A correctly optimized score network $s_{\chi }\left(r,t\right)$ allows samples from the joint distribution $q_{\psi }\left(z\right)$ to be obtained through simulation of Equation (5). Similarly, through the simulation of Equation (8), a *conditional* score network $s_{\chi }\left(r^{A_{1}},t\;|\;z^{A_{2}}\right)$ allows for sampling from $q_{\psi }\left(z^{A_{1}}\;|\;z^{A_{2}}\right)$. In Section 4.1, we extend the guidance mechanisms used in classical diffusion models to allow multimodal conditional generation. A naïve alternative is to rely on the unconditional score network $s_{\chi }\left(r,t\right)$ for the conditional generation task by casting it as an *in-painting* objective. Intuitively, any missing modality could be recovered in the same way that a unimodal diffusion model can recover masked information. In Section 4.3, we discuss the implicit assumptions underlying in-painting from an information-theoretic perspective and argue that such assumptions are difficult to satisfy in the context of multimodal data. This intuition is corroborated by ample empirical evidence, where our method consistently outperforms alternatives.

### 4.1. Multi-Time Diffusion

We propose a modification to the classifier-free guidance technique [48] to learn a score network that can generate conditional and unconditional samples from any subset of modalities. Instead of training a separate score network for each possible combination of conditional modalities, which is computationally infeasible, we use a single architecture that accepts all modalities as inputs and a *multi-time vector* $\tau =[t_{1},\ldots ,t_{M}]$. The multi-time vector serves two purposes: it is both a conditioning signal and the time at which we observe the diffusion process.

**Training:** Learning the conditional score network relies on randomization. As discussed in Section 3, we consider an arbitrary partitioning of all modalities in two disjoint sets, $A_{1}$ and $A_{2}$; set $A_{2}$ contains randomly selected conditioning modalities, while the remaining modalities belong to set $A_{1}$. During training, the parametric score network estimates $\nabla \log(q\left(r,t\;|\;z^{A_{2}}\right))$, whereby set $A_{2}$ is randomly chosen at every step. This is achieved by the *masked diffusion process* from Equation (7), which only diffuses modalities in $A_{1}$. More formally, the score network input is $R_{t}=C\left(R_{t}^{A_{1}},Z^{A_{2}}\right)$, along with a multi-time vector $\tau \left(A_{1},t\right)=t\left[\begin{array}{c} 1\left(1\in A_{1}\right),\ldots ,1\left(M\in A_{1}\right) \end{array}\right]$. As a follow-up of the example in Section 3, given $A_{1}=\left\{1,3,5\right\}$ such that $X^{A_{1}}=\left\{X^{1},X^{3},X^{5}\right\}$ and $A_{2}=\left\{2,4,6\right\}$ such that $X^{A_{2}}=\left\{X^{2},X^{4},X^{6}\right\}$, we have $\tau \left(A_{1},t\right)=\left[t,0,t,0,t,0\right]$.

More precisely, the algorithm for multi-time diffusion training (see Appendix A for the pseudo-code) proceeds as follows. At each step, a set of conditioning modalities $A_{2}$ is sampled from a predefined distribution ν, where $\nu \left(\emptyset \right)\overset{\mathrm{def}}{=}\mathrm{Pr}\left(A_{2}=\emptyset \right)=d$ and $\nu \left(U\right)\overset{\mathrm{def}}{=}\mathrm{Pr}\left(A_{2}=U\right)=\left(1-d\right)\;/\;\left(2^{M}-1\right)$ with U∈P({1,…,M})∖∅, where P({1,…,M}) is the powerset of all modalities. The corresponding set $A_{1}$ and mask $m(A_{1})$ are constructed, and a sample *X* is drawn from the training dataset. The corresponding latent variables $Z^{A_{1}}={\left\{e_{\psi }^{i}\left(X^{i}\right)\right\}}_{i\in A_{1}}$ and $Z^{A_{2}}={\left\{e_{\psi }^{i}\left(X^{i}\right)\right\}}_{i\in A_{2}}$ are computed using the pretrained encoders and a diffusion process starting from $R_{0}=C\left(Z^{A_{1}},Z^{A_{2}}\right)$ is simulated for a randomly chosen diffusion time *t* using the conditional forward SDE with the mask $m(A_{1})$. The score network is then fed the current state $R_{t}$ and multi-time vector $\tau (A_{1},t)$ and the difference between the score network’s prediction and the true score is computed while applying mask $m(A_{1})$. The score network parameters are updated using stochastic gradient descent, and this process is repeated for a total of *L* training steps. Clearly, when $A_{2}=\emptyset$, training proceeds the same as for an unmasked diffusion process, as mask $m(A_{1})$ allows all of the latent variables to be diffused.

**Conditional generation:** Any valid numerical integration scheme for Equation (8) can be used for conditional sampling (see Appendix A for an implementation using the Euler–Maruyama integrator). First, conditioning modalities in set $A_{2}$ are encoded into the corresponding latent variables $z^{A_{2}}={\left\{e^{j}\left(x^{j}\right)\right\}}_{j\in A_{2}}$. Then, numerical integration is performed with a step size of Δt=T/N, starting from initial conditions $R_{0}=C\left(R_{0}^{A_{1}},z^{A_{2}}\right)$ with $R_{0}^{A_{1}}∼\rho \left(r^{A_{1}}\right)$. At each integration step, the score network $s_{\chi }$ is fed the current state of the process and the multi-time vector $\tau (A_{1},\cdot )$. Before updating the state, the masking is applied. Finally, the generated modalities are obtained thanks to the decoders as ${\hat{X}}^{A_{1}}={\left\{d_{\theta }^{j}\left(R_{T}^{j}\right)\right\}}_{j\in A_{1}}$. Inference time conditional generation is not randomized; the conditioning modalities are the ones that are available, whereas those remaining are the ones we wish to generate.

Any-to-any multimodality has been recently studied through the composition of modality-specific diffusion models [49] by designing cross-attention and training procedures that allow for arbitrary conditional generation. This work by Tang et al. [49] relies on latent interpolation of input modalities, which is akin to mixture models, and uses it as conditioning signal for individual diffusion models. This is substantially different from the joint nature of the multimodal latent diffusion we present in our work; instead of forcing entanglement through cross-attention between score networks, our model relies on a joint diffusion process whereby modalities naturally co-evolve according. Another recent work [50], targeted multimodal conversational agents, wherein the strong underlying assumption is to consider one modality, i.e., text, as a guide for the alignment and generation of other modalities. Even if conversational objectives are orthogonal to our work, techniques akin to instruction-following for cross-generation are an interesting illustration of the powerful capabilities of in-context learning on the part of LLMs [51,52].

### 4.2. Multimodal Interaction

MLD treats the latent spaces of each modality as variables that evolve differently through the diffusion process according to a multi-time vector. The masked multi-time training enables the model to learn the score of all the combinations of conditionally diffused modalities, using the frozen modalities as the conditioning signal through a randomized scheme. By learning the score function of the diffused modalities at different time steps, the score model captures the correlation between the modalities.

At test time, the diffusion time of each modality is chosen so as to modulate its influence on the generation. For joint generation, the model uses the unconditional score, which corresponds to using the same diffusion time for all modalities. Thus, all the modalities influence each other equally. This ensures that the modality interaction information is faithful to the information characterizing the observed data distribution. The model can also generate modalities conditionally using the conditional score by freezing the conditioning modalities during the reverse process. The frozen state is similar to the final state of the revere process, where information is not perturbed; thus, the influence of the conditioning modalities is maximal. Subsequently, the generated modalities reflect the necessary information from the conditioning modalities and achieve the desired correlation.

### 4.3. In-Painting and Its Implicit Assumptions

Under certain assumptions, given an unconditional score network $s_{\chi }\left(r,t\right)$ that approximates the true score ∇logq(r,t), it is possible to obtain a conditional score network $s_{\chi }\left(r^{A_{1}},t\;|\;z^{A_{2}}\right)$ to approximate $\nabla \log q(r^{A_{1}},t\;|\;z^{A_{2}})$. We start by observing the equality

(9) $$q\left(r^{A_{1}},t\;|\;z^{A_{2}}\right)=\int q\left(C\left(r^{A_{1}},r^{A_{2}}\right),t\;|\;z^{A_{2}}\right)dr^{A_{2}}=\int \frac{q(z^{A_{2}}\;|\;C\left(r^{A_{1}},r^{A_{2}}\right),t)}{q_{\psi }\left(z^{A_{2}}\right)}q\left(C\left(r^{A_{1}},r^{A_{2}}\right),t\right)dr^{A_{2}},$$

where, with a slight abuse of notation, we indicate with $q(z^{A_{2}}\;|\;C\left(r^{A_{1}},r^{A_{2}}\right),t)$ the density associated with the event; the portion corresponding to $A_{2}$ of the latent variable *Z* is equal to $z^{A_{2}}$, given that the whole diffused latent $R_{t}$ at time *t* is equal to $C(r^{A_{1}},r^{A_{2}})$. In the literature, the quantity $q(z^{A_{2}}\;|\;C\left(r^{A_{1}},r^{A_{2}}\right),t)$ is typically approximated by dropping its dependency on $r^{A_{1}}$. This approximation can be used to manipulate Equation (9) as $q\left(r^{A_{1}},t\;|\;z^{A_{2}}\right)≃\int q\left(r^{A_{2}},t\;|\;z^{A_{2}}\right)q\left(r^{A_{1}},t|r^{A_{2}},t\right)dr$. Further, Monte Carlo approximations [32,53] of the integral allows for implementation of a practical scheme in which an approximate conditional score network is used to generate conditional samples. This approach, known in the literature as *in-painting*, provides high quality results in several *unimodal* application domains [32,53].

By fixing $r^{A_{1}},r^{A_{2}}$, the KL divergence between $q(z^{A_{2}}\;|\;C\left(r^{A_{1}},r^{A_{2}}\right),t)$ and $q(z^{A_{2}}\;|\;r^{A_{2}},t)$ quantifies the discrepancy between the true and approximated conditional probabilities. Similarly, the expected KL divergence

(10) $$\begin{array}{c} \Delta =\int q\left(r,t\right)\mathrm{KL}[q\left(z^{A_{2}}\;|\;C\left(r^{A_{1}},r^{A_{2}}\right),t\right)\;|\;\;|\;q\left(z^{A_{2}}\;|\;r^{A_{2}},t\right)]dr \end{array}$$

provides information about the average discrepancy. Simple manipulations allow this to be recast as a discrepancy in terms of the mutual information $\Delta =I\left(Z^{A_{2}};R_{t}^{A_{1}},R_{t}^{A_{2}}\right)-I\left(Z^{A_{2}};R_{t}^{A_{2}}\right)$. Information about $Z^{A_{2}}$ is contained in $R_{t}^{A_{2}}$, as the latter is the result of a diffusion with the former as initial conditions, corresponding to the Markov chain $R_{t}^{A_{2}}\to Z^{A_{2}}$, and in $R_{t}^{A_{1}}$ through the Markov chain $Z^{A_{2}}\to Z^{A_{1}}\to R_{t}^{A_{1}}$. The positive quantity Δ is close to zero whenever the rate of loss of information with respect to the initial conditions is similar for the two subsets $A_{1}$ and $A_{2}$. In other terms, Δ≃0 whenever the portion $R_{t}^{A_{2}}$ of the whole $R_{t}$ is a sufficient statistic for $Z^{A_{2}}$.

The assumptions underlying the approximation are in general not valid in the case of multimodal learning, where the robustness to stochastic perturbations of latent variables corresponding to the various modalities can vary greatly. In Appendix B, our claims are empirically supported by ample analysis performed on real data showing that our multi-time diffusion approach consistently outperforms in-painting.

## 5. Experiments

We compared our MLD method to MVAE [21], MMVAE [22], MOPOE [23], Hierarchical Genertive Model (NEXUS) [26], Multi-view Total Correlation Autoencoder (MVTCAE) [27], and MMVAE+ [29], re-implementing all competitors in the same code base as our method and selecting their best hyperparameters as indicated by the authors (see Appendix D for more details). For a fair comparison, we used the same encoder/decoder architecture for all models. For MLD, the score network was implemented using a simple stacked multilayer perceptron (MLP) with skip connections (see Appendix A for more details). MLD was also contrasted with multimodal diffusion-based approaches: [35] in Appendix B and [36] in Section 5.5.

**Evaluation metrics:** *Coherence* was measured as in [22,23,29], using pretrained classifiers on the generated data and checking the consistency of their outputs. *Generative quality* was computed using the Fréchet Inception Distance (FID) [54] and Fréchet Audio Distance (FAD) [55] scores for images and audio, respectively. Full details on the metrics are included in Appendix C. All results were averaged over five seeds. We report the standard deviations in Appendix E.

**Results:** Overall, MLD largely outperformed the alternatives from the literature in terms of both coherence and generative quality. The VAE-based models suffered from the coherence–quality tradeoff as well as from modality collapse for highly heterogeneous datasets. We proceed to show this on several standard benchmarks from the multimodal VAE-based literature; see Appendix C for details on the datasets.

### 5.1. MNIST-SVHN

**Figure 2:**
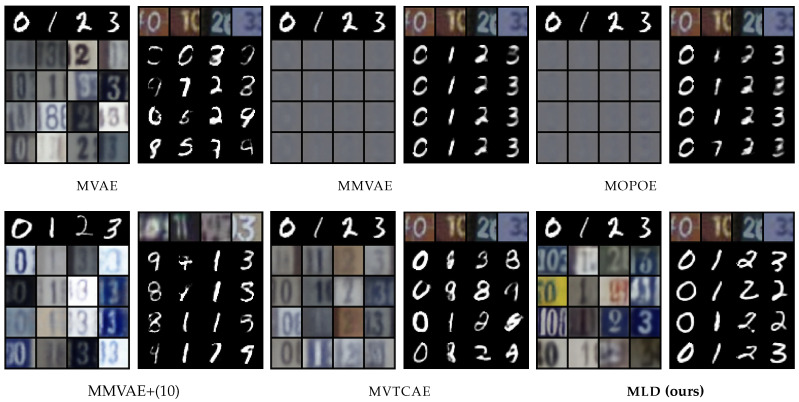
Qualitative results for **MNIST-SVHN**. For each model, we report MNIST to SVHN conditional generation on the left and SVHN to MNIST conditional generation on the right. The conditioning modality is illustrated by the first row, with the generated samples below.

The first dataset we consider is **MNIST-SVHN** [22], where the two modalities differ in complexity. High variability, noise, and ambiguity make attaining good coherence for the SVHN modality a challenging task. Overall, MLD outperforms all VAE-based alternatives in terms of coherency, especially in terms of joint generation and conditional generation of MNIST given SVHN (see Table 1). The mixture models, MMVAE and MOPOE, suffer from modality collapse (poor SVHN generation), whereas the product-of-experts models MVAE and MVTCAE generate better-quality samples at the expense of SVHN to MNIST conditional coherence. Joint generation is poor for all VAE models. Interestingly, these models also fail at SVHN self-reconstruction, which we discuss in Appendix E. MLD also achieves the best performance in terms of generation quality, as confirmed by qualitative results (Figure 2) showing, for example, how MLD conditionally generates multiple SVHN digits within one sample given the input MNIST image, whereas the other methods fail to do so.

### 5.2. MHD

The Multimodal Handwritten Digits dataset (**MHD**) [26] contains gray-scale images of digits, the motion trajectory of the handwriting, and the sounds of the spoken digits. In our experiments, we did not use the label as a fourth modality. While the images and trajectories share a good amount of information, the sound modality contains a great deal more modality-specific variation. Consequently, both conditional generation involving the sound modality and joint generation represent challenging tasks. Coherency-wise, (Table 2) MLD outperforms all the competitors, with the biggest difference seen in joint generation and generation from sound to other modalities. On the latter task, MVTCAE performs better than other competitors, but is still worse than MLD. MLD dominates the alternatives in terms of generation quality (Table 3). This is true both for image and sound modalities, for which some VAE-based models struggle to produce high-quality results, demonstrating the limitation of these methods in handling highly heterogeneous modalities. MLD, on the other hand, achieves high generation quality for all modalities, possibly due to the independent training of the autoencoders avoiding interference.

### 5.3. POLYMNIST

**Figure 3:**
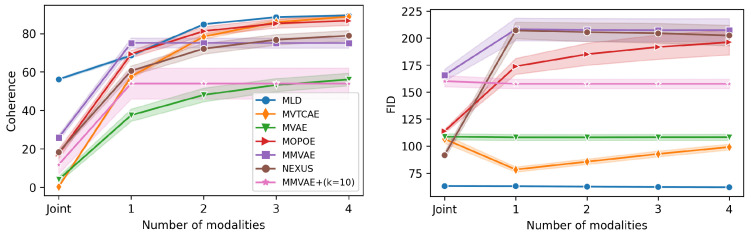
Performance results for **POLYMNIST** as a function of the number of inputs. (**Right**): Generative coherence (% ↑). (**Left**): Generative quality in terms of FID (↓). We report the average performance following the leave-one-out strategy (see Appendix C).

**Figure 4:**
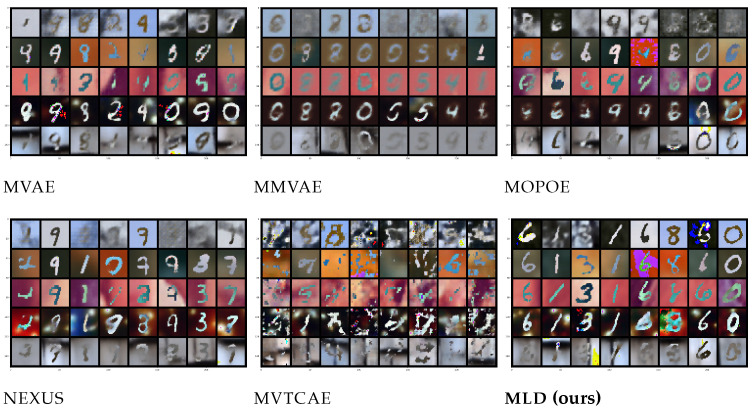
Joint generation qualitative results for **POLYMNIST** across the five modalities.

The **POLYMNIST** dataset [23] consists of five modalities synthetically generated using MNIST digits and varying the background images. The homogeneous nature of the modalities is expected to mitigate gradient conflict issues in VAE-based models and consequently reduce modality collapse. However, MLD still outperforms all alternatives, as shown in Figure 3 and Figure 4. Concerning generation coherence, MLD achieves the best performance in all cases, with the one exception of a single observed modality. On the qualitative performance side, not only is MLD superior to all alternatives, its results are stable when more modalities are considered, a capability that not all competitors share.

### 5.4. CUB

**Figure 5:**
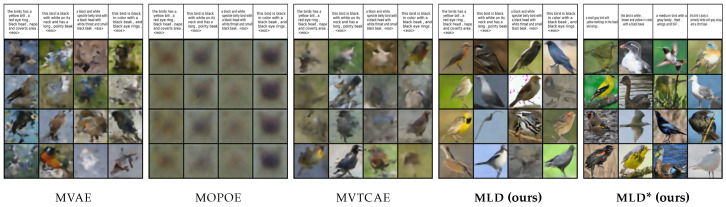
Qualitative results on the **CUB** dataset, with the caption used as the condition to generate the bird images. MLD* denotes the version of our method using a powerful image autoencoder.

Next, we explored the Caltech Birds **CUB** [22] dataset, following the experimental protocol in [24] using real bird images instead of ResNet-features as in [22]. Figure 5 presents qualitative results for caption-to-image conditional generation. MLD is the only model capable of generating bird images with convincing coherence. Clearly, none of the VAE-based methods is able to achieve sufficient caption-to-image conditional generation quality using the same simple autoencoder architecture. Note that an image autoencoder with larger capacity considerably improves the generative performance of MLD, suggesting that careful engineering applied to modality-specific autoencoders is a promising avenue for future work. We report quantitative results in Appendix E, where we show the generation quality FID metric. Due to the unavailability of the labels in this dataset, the coherence evaluation performed with the previous datasets was not possible. Thus, we resorted to CLIP-Score (CLIP-S) [56], an image-captioning metric. Despite its limitations for the considered dataset [57], CLIP-S shows that MLD outperforms all competitors.

### 5.5. CelebAMask-HQ

**Figure 6:**
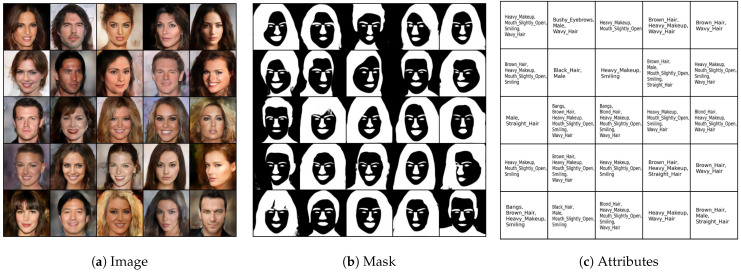
Joint (unconditional) generation: qualitative results of **MLD** on CelebAMask-HQ.

**Figure 7:**
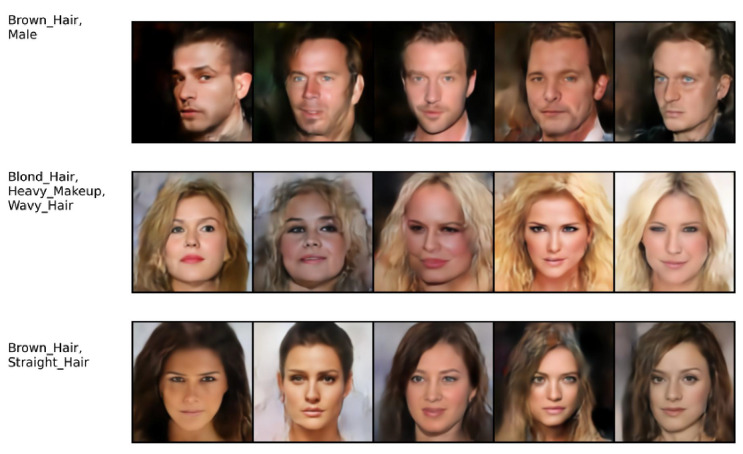
(Attributes → Image). Conditional generation of **MLD** on CelebAMask-HQ. The first column on the left presents the conditioning modalities, while several conditionally generated samples are displayed on the right.

**Figure 8:**
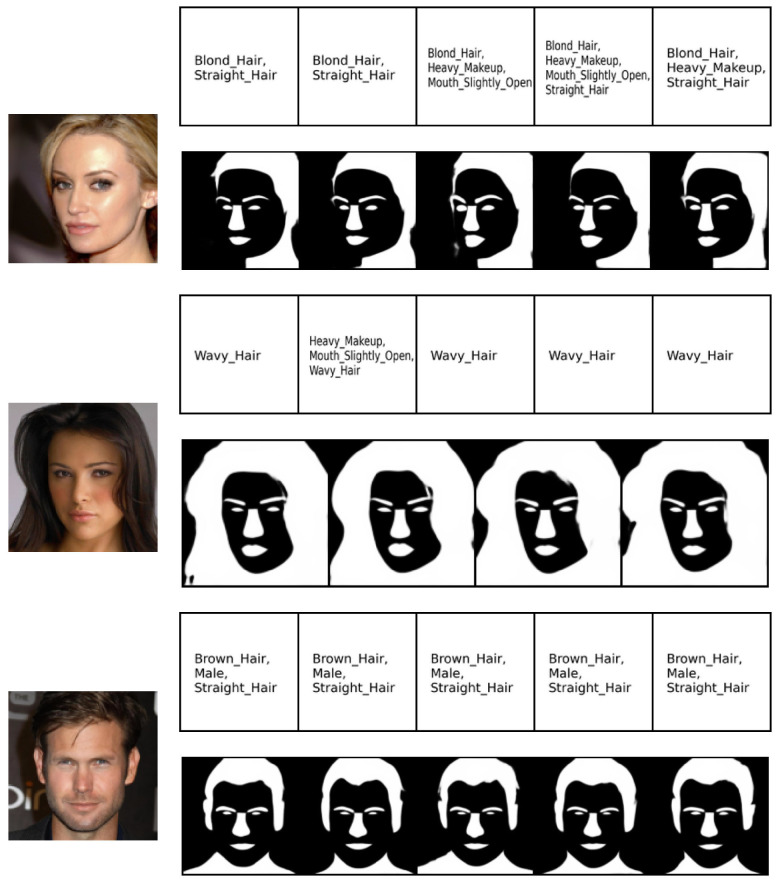
(Image → Attribute, Mask). Conditional generation of **MLD** on CelebAMask-HQ. The first column on the left presents the conditioning modalities, while several conditionally generated samples are displayed on the right.

**Figure 9:**
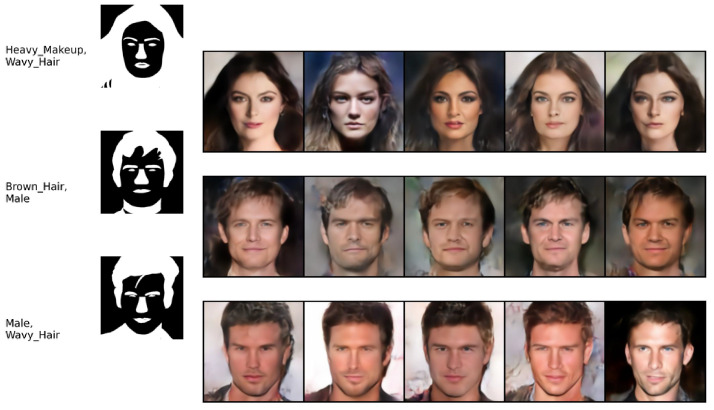
(Attributes, Mask → Image). Conditional generation of **MLD** on CelebAMask-HQ. The two columns on the left present the conditioning modalities, while several conditionally generated samples are displayed on the right.

Finally, we considered the CelebAMask-HQ dataset [58], which consists of three modalities: face images, each having a segmentation mask and text attributes. We followed the same experimental protocol as in [36], including the autoencoder base architecture. The image generation quality was evaluated in terms of FID score. The attributes and the mask, both having binary values, were evaluated against the ground truth in terms of the F1 score. The competitors’ performance results are reported from [36]. The quantitative results in Table 4 show that MLD outperforms the competitors in terms of generation quality. Our method achieves the best F1 score in generation of the attribute modalities given the image and mask modalities. In mask generation, MOPOE and MVTCAE achieve the best performance, with MLD achieving the second-best performance in mask generation conditioning on both the image and attribute modalities. Overall, MLD stands out with the best image quality generation, while being on par with the competition in terms of mask and attribute generation coherence. Figure 6 shows the qualitative results for MLD on the joint generation task. It can be observed that our method succeeds at generating all three modalities with high coherence and quality. The same observation is valid for the conditional generation tasks (see Figure 7, Figure 8 and Figure 9).

## 6. Conclusions and Limitations

We have presented a new multimodal generative model, Multimodal Latent Diffusion (MLD), to address the well known coherence–quality tradeoff that is inherent in existing multimodal VAE-based models. MLD uses a set of independently trained unimodal deterministic autoencoders. The generative properties of our model stem from a masked diffusion process that operates on latent variables. In addition, we have developed a new multi-time training method to learn the conditional score network for multimodal diffusion. An extensive experimental campaign on various real-life datasets provides compelling evidence of the effectiveness of MLD for multimodal generative modeling. In all scenarios, including cases with loosely correlated modalities and high-resolution datasets, MLD consistently outperforms state-of-the-art alternatives. A limitation of our approach stems from the simple nature of encoder/decoder architectures. Focusing on more specialized, complex, and tailor-made encoder/decoder architectures might be necessary when moving to higher-resolution data. As for all generative models, ours could be misused to produce misinformation. We believe, however, that the benefits of multimodal generative models outweigh their potential misuses.

## Figures and Tables

**Table 1:** Generation coherence and quality for **MNIST-SVHN** (M: MNIST, S: SVHN). The generation quality is measured in terms of the Fréchet Modality Distance (FMD) for MNIST and FID for SVHN. We report both joint and conditional generation performance results. Bold and underlined numbers indicate the best and second best scores respectively.

Models	Coherence (%↑)			Quality (↓)			
	Joint	M→S	S→M	Joint (M)	Joint(S)	M→S	S→M
MVAE	38.19	48.21	28.57	13.34	68.9	$\underline{68.0}$	13.66
MMVAE	37.82	11.72	67.55	25.89	146.82	393.33	53.37
MOPOE	39.93	12.27	68.82	20.11	129.2	373.73	43.34
NEXUS	40.0	16.68	$\underline{70.67}$	13.84	98.13	281.28	53.41
MVTCAE	$\underline{48.78}$	$\underline{81.97}$	49.78	$\underline{12.98}$	52.92	69.48	$\underline{13.55}$
MMVAE+	17.64	13.23	29.69	26.60	121.77	240.90	35.11
MMVAE+ (K = 10)	41.59	55.3	56.41	19.05	67.13	75.9	18.16
**MLD (ours)**	85.22	83.79	79.13	3.93	$\underline{56.36}$	57.2	3.67

**Table 2:** Generation coherence (%) for **MHD** (higher is better). Line above refers to the generated modality, while the subset of observed modalities is presented below. Bold and underlined numbers indicate the best and second best scores respectively.

Models	Joint	I (Image)			T (Trajectory)			S (Sound)		
		T	S	T,S	I	S	I,S	I	T	I,T
MVAE	37.77	11.68	26.46	28.4	95.55	26.66	96.58	58.87	10.76	58.16
MMVAE	34.78	99.7	69.69	84.74	$\underline{99.3}$	85.46	92.39	49.95	50.14	50.17
MOPOE	48.84	$\underline{99.64}$	68.67	$\underline{99.69}$	99.28	$\underline{87.42}$	99.35	50.73	51.5	56.97
NEXUS	26.56	94.58	$\underline{83.1}$	95.27	88.51	76.82	93.27	70.06	75.84	89.48
MVTCAE	42.28	99.54	72.05	99.63	99.22	72.03	$\underline{99.39}$	$\underline{92.58}$	$\underline{93.07}$	$\underline{94.78}$
MMVAE+	41.67	98.05	84.16	91.88	97.47	81.16	89.31	64.34	65.42	64.88
MMVAE+ (K = 10)	42.60	99.44	89.75	94.7	99.44	89.58	95.01	87.15	87.99	87.57
**MLD (ours)**	98.34	99.45	$\underline{88.91}$	99.88	99.58	$\underline{88.92}$	99.91	97.63	97.7	98.01

**Table 3:** Generation quality for **MHD** in terms of FMD for image and trajectory modalities and FAD for the sound modality (lower is better). Bold and underlined numbers indicate the best and second best scores respectively.

Models	I (Image)				T (Trajectory)				S (Sound)			
	Joint	T	S	T,S	Joint	I	S	I,S	Joint	I	T	I,T
MVAE	$\underline{94.9}$	93.73	92.55	91.08	39.51	20.42	38.77	19.25	14.14	$\underline{14.13}$	14.08	14.17
MMVAE	224.01	22.6	789.12	170.41	16.52	0.5	30.39	6.07	22.8	22.61	23.72	23.01
MOPOE	147.81	16.29	838.38	15.89	$\underline{13.92}$	$\underline{0.52}$	33.38	0.53	18.53	24.11	24.1	23.93
NEXUS	281.76	116.65	282.34	117.24	18.59	6.67	33.01	7.54	$\underline{13.99}$	19.52	18.71	16.3
MVTCAE	121.85	$\underline{5.34}$	$\underline{54.57}$	$\underline{3.16}$	19.49	0.62	$\underline{13.65}$	0.75	15.88	14.22	$\underline{14.02}$	$\underline{13.96}$
MMVAE+	97.19	2.80	128.56	114.3	22.37	1.21	21.74	15.2	16.12	17.31	17.92	17.56
MMVAE+ (K = 10)	85.98	1.83	70.72	62.43	21.10	1.38	8.52	7.22	14.58	14.33	14.34	14.32
MLD	7.98	1.7	4.54	1.84	3.18	0.83	2.07	$\underline{0.6}$	2.39	2.31	2.33	2.29

**Table 4:** Quantitative results on the CelebAMask-HQ dataset. Performance is measured in terms of the FID (↓) and F1 score (↑). The first row shows the generated modality, while the second row shows the modalities used as conditions. Supervised classifier designates a classifier performance to predict the attributes or the mask from an image. Bold numbers indicate the best scores.

Models	Attributes		Image				Mask	
	Img + Mask	Img	Att + Mask	Mask	Att	Joint	Img + Att	Img
	F1	F1	FID	FID	FID	FID	F1	F1
SBM-RAE [36]	0.62	0.6	84.9	86.4	85.6	84.2	0.83	0.82
SBM-RAE-C [36]	0.66	0.64	83.6	82.8	83.1	84.2	0.83	0.82
SBM-VAE [36]	0.62	0.58	81.6	81.9	78.7	79.1	0.83	0.83
SBM-VAE-C [36]	0.69	0.66	82.4	81.7	76.3	79.1	0.84	0.84
MOPOE	0.68	**0.71**	114.9	101.1	186.8	164.8	0.85	**0.92**
MVTCAE	0.71	0.69	94	84.2	87.2	162.2	**0.89**	0.89
MMVAE+	0.64	0.61	133	97.3	153	103.7	0.82	0.89
Supervised classifier		0.79					0.94	
**MLD (ours)**	0.72	0.69	52.75	51.73	53.09	54.27	0.87	0.87

**Figure A1:**
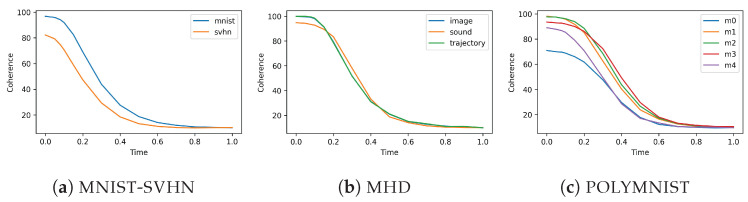
Coherence as a function of the diffusion process time for three datasets. Diffusion perturbation is applied on the modalities’ latent space after element-wise normalization.

**Figure A2:**
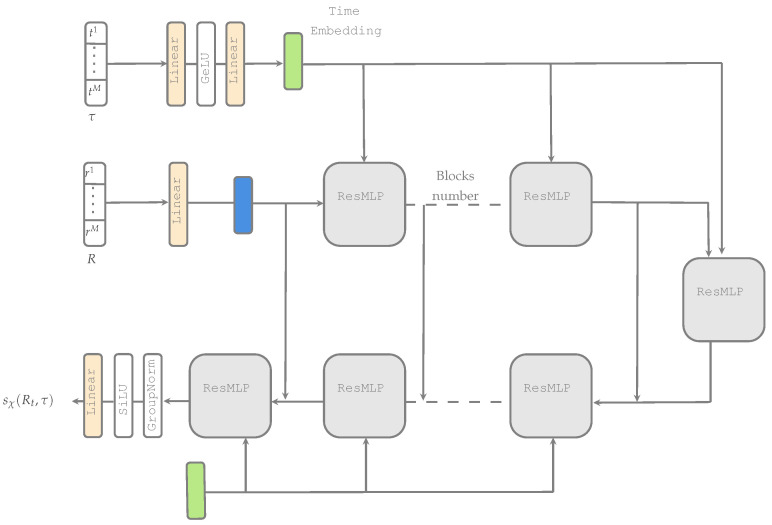
Score network $s_{\chi }$ architecture used in our MLD implementation. The residual MLP block architecture is shown in Figure A3.

**Figure A3:**
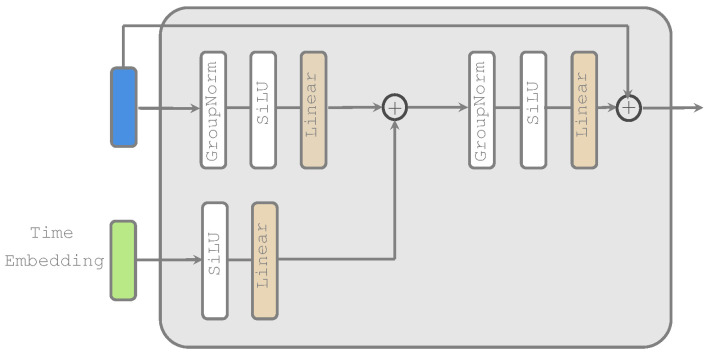
Architecture of the ResMLP block.

**Figure A4:**
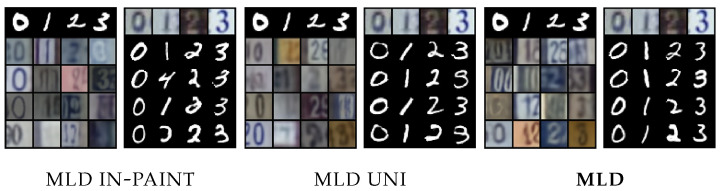
Qualitative results for **MNIST-SVHN**. For each model, we report MNIST-to-SVHN conditional generation on the left and SVHN-to-MNIST conditional generation on the right.

**Figure A5:**
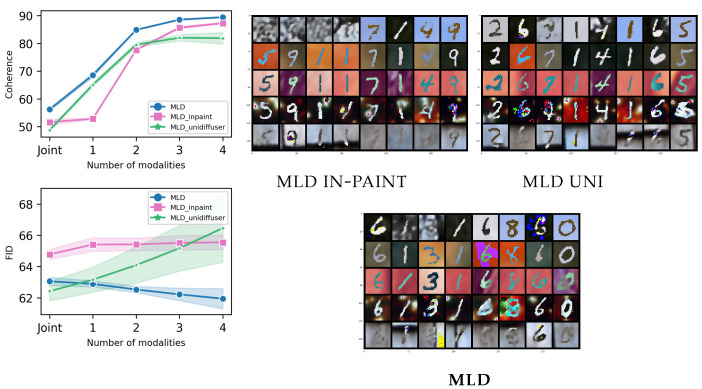
Results for the **POLYMNIST** dataset. (**Left**): a comparison of the generative coherence (% ↑) and quality in terms of FID (↓)) as a function of the number of modality inputs. We report the average performance following the leave-one-out strategy (see Appendix C). (**Right**): qualitative results for joint generation of the five modalities.

**Figure A6:**
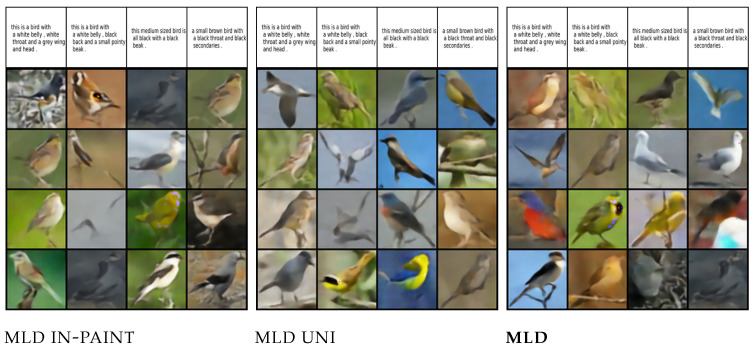
Qualitative results on the **CUB** dataset. Captions were used as the condition to generate the bird images.

**Figure A7:**
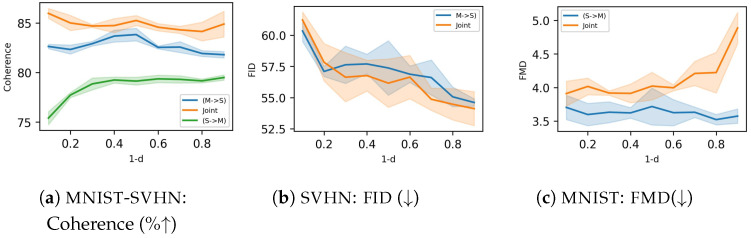
Results of the ablation study for the randomization parameter *d* on the **MNIST-SVHN** dataset.

**Figure A8:**
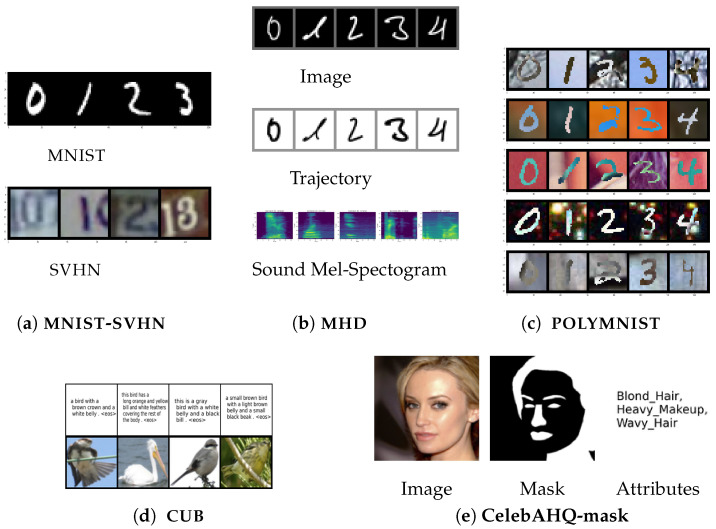
Illustrative example of the datasets used for evaluation.

**Figure A9:**
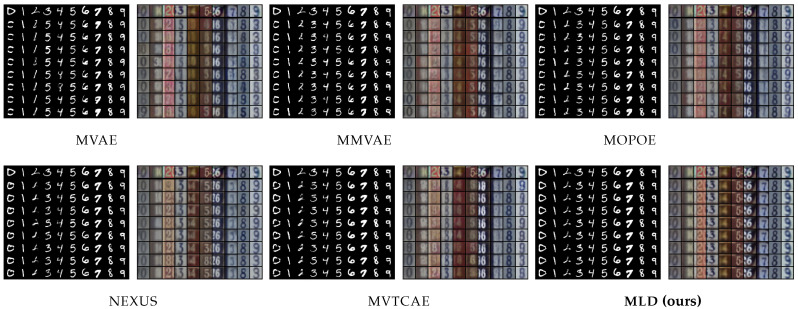
Self-generation qualitative results for **MNIST-SVHN**. For each model, we report MNIST-to-MNIST conditional generation on the left and SVHN-to-SVHN conditional generation on the right.

**Figure A10:**
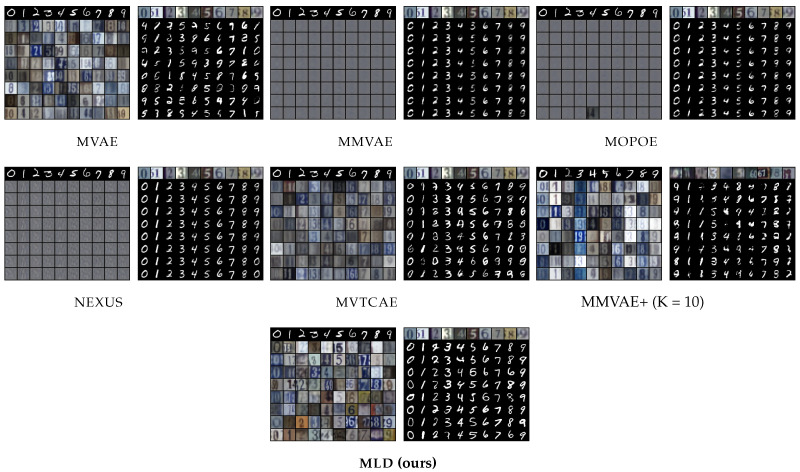
Additional qualitative results for **MNIST-SVHN**. For each model, we report MNIST-to-SVHN conditional generation on the left and SVHN-to-MNIST conditional generation on the right.

**Figure A11:**
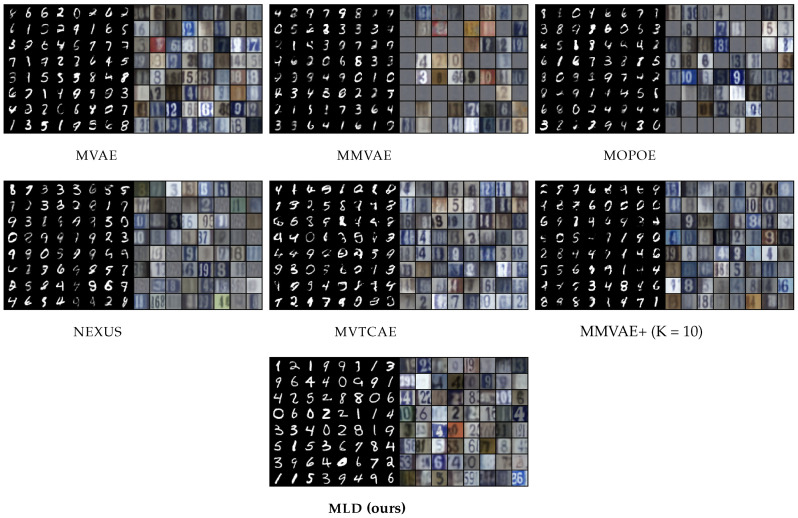
Qualitative results for **MNIST-SVHN** joint generation.

**Figure A12:**
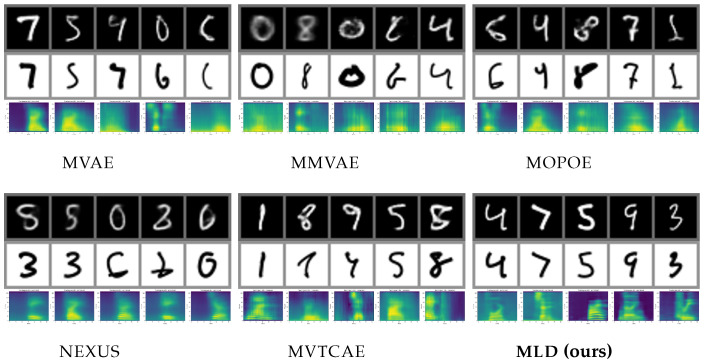
Joint generation qualitative results for **MHD**. The three modalities were randomly generated simultaneously. **Top row**: image; **Middle row**: trajectory vector converted into image; **Bottom row**: sound mel-spectrogram).

**Figure A13:**
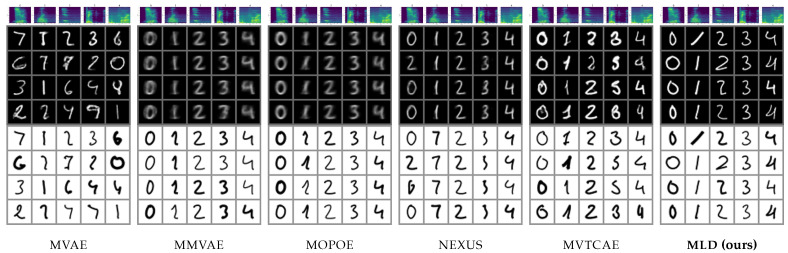
Sound-to-image and trajectory conditional generation qualitative results for **MHD**. For each model, the **Top row** reports the sound mel-spectrograms of the digits {0,1,2,3,4} from left to right and the **Lower rows** report the generated image and trajectory samples.

**Figure A14:**
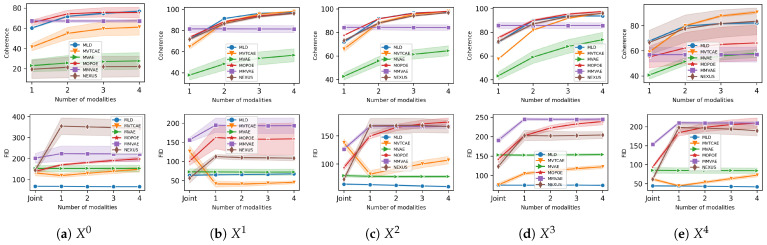
**Top:** Generation coherence (%) for **POLYMNIST** (higher is better). **Bottom:** Generation quality (FID) (lower is better). We report the average *leave-one-out* performance as a function of the number of observed modalities for each modality $X^{i}$. *Joint* refers to random generation of the five modalities simultaneously.

**Figure A15:**
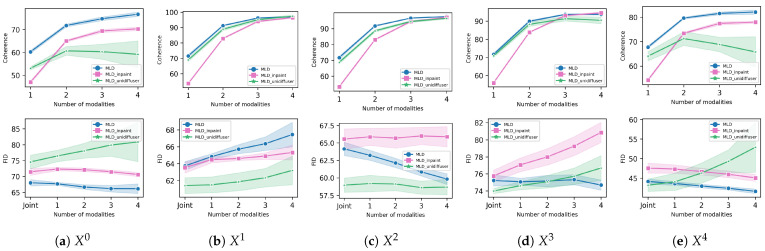
**Top:** Generation coherence (%) for **POLYMNIST** (higher is better). **Bottom:** Generation quality (FID) (lower is better). We report the average *leave-one-out* performance as a function of the number of observed modalities for each modality $X^{i}$. *Joint* refers to random generation of the five modalities simultaneously.

**Figure A16:**
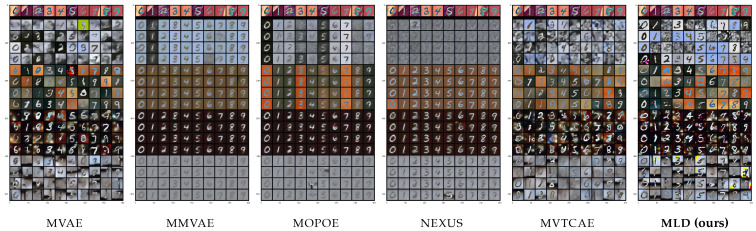
Conditional generation qualitative results for **POLYMNIST**. Modality $X^{2}$ (first row) is used as the condition to generate the four remaining modalities (the rows below).

**Figure A17:**
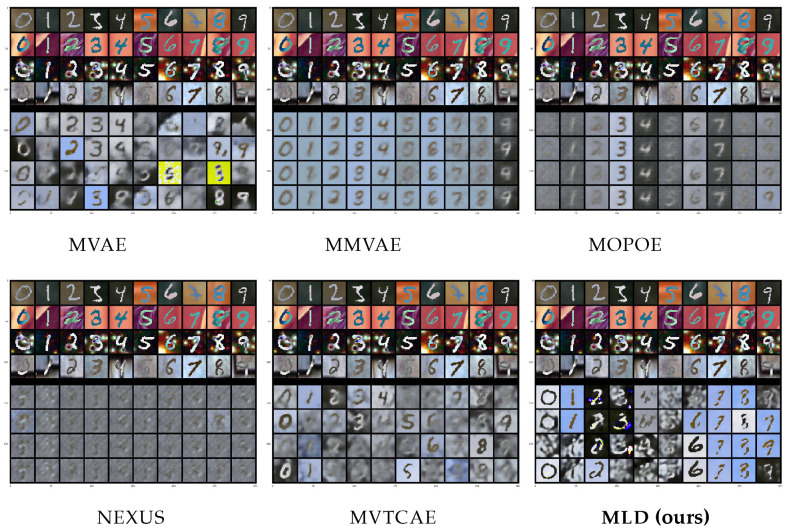
Conditional generation qualitative results for **POLYMNIST**. The subset of modalities $X^{1},X^{2},X^{3},X^{4}$ (first four rows) are used as the condition to generate modality $X^{0}$ (the rows below).

**Figure A18:**
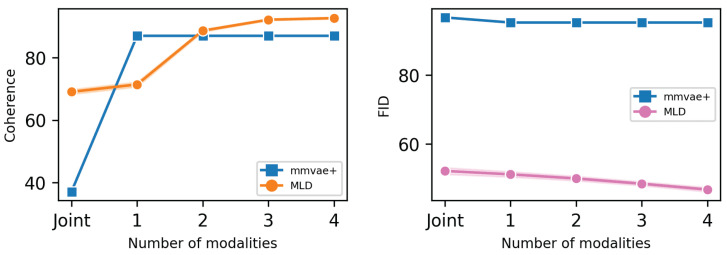
Results for the POLYMNIST dataset. Left: Comparison of the generative coherence (↑) and quality in terms of FID (↓) as a function of the number of inputs.

**Figure A19:**
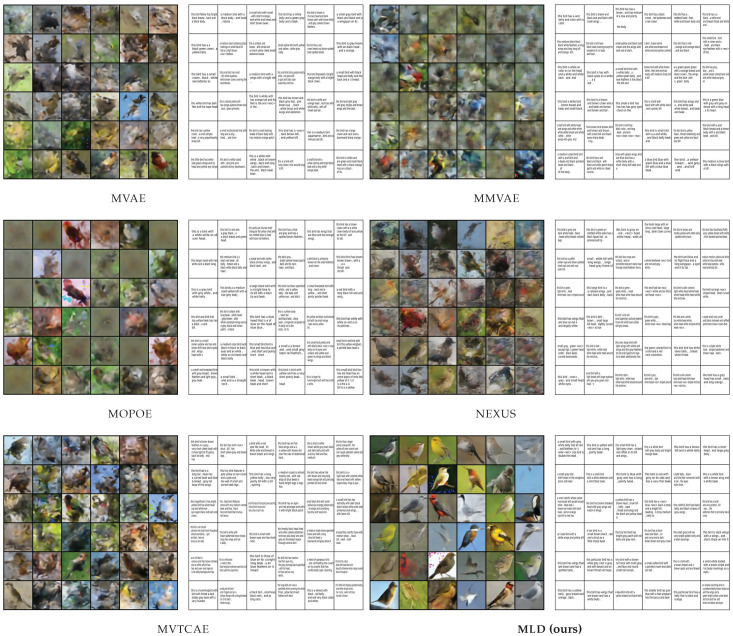
Qualitative results for joint generation on the **CUB** dataset (Better viewed zoomed).

**Figure A20:**
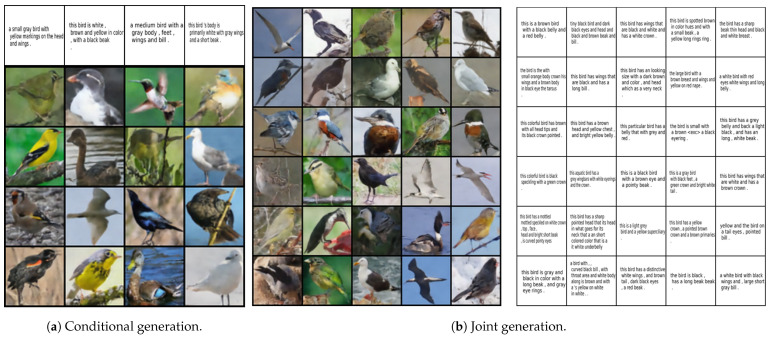
Qualitative results of MLD* on the **CUB** dataset with powerful image autoencoder (Better viewed zoomed).

**Figure A21:**
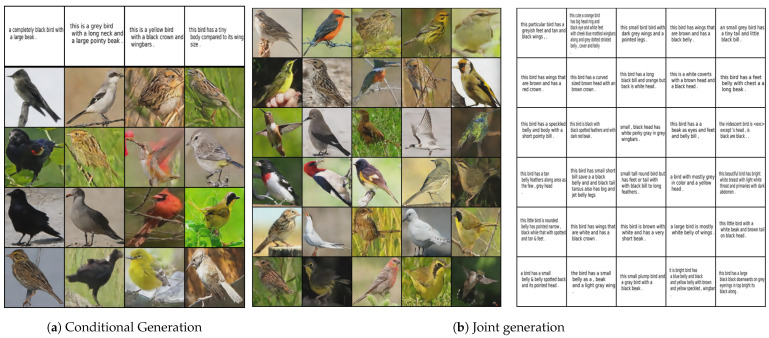
Qualitative results of MLD* on the **CUB** dataset with 128 × 128 resolution images and powerful image autoencoder (Better viewed zoomed).

**Figure A22:**
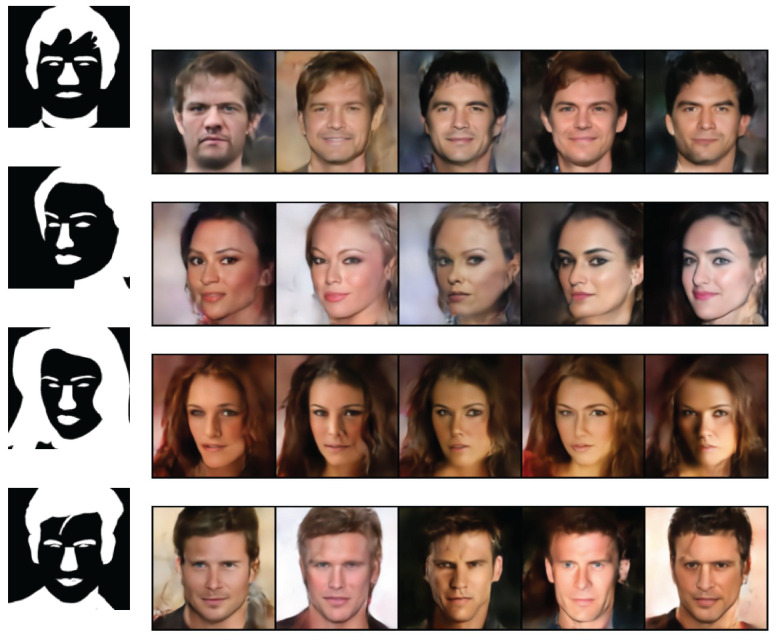
(Mask → Image) Conditional generation of **MLD** on the CelebAMask-HQ dataset.

## Data Availability

All used datasets are publicly available. Our code is available at https://github.com/MustaphaBounoua/MLD.

## Appendix A. Diffusion in the Multimodal Latent Space

In this appendix, we provide additional technical details of MLD. We first discuss a naive approach based on *in-painting* which uses only the unconditional score network for both joint and conditional generation. We also discuss an alternative training scheme based on a work from the caption-text translation literature [35]. Finally, we provide extra technical details for the score network architecture and sampling technique.

### Appendix A.1. Modality Auto-Encoders

Each of the deterministic autoencoders used in the first stage of MLD uses a vector latent space with no size constraints. Instead, VAE-based models generally require the latent space of each individual VAE to be exactly the same size to allow for the definition of a joint latent space.

In our approach, the modality-specific latent spaces are *normalized* prior to concatenation using the element-wise mean and standard deviation. In practice, we use the statistics retrieved from the first training batch, which we found to provide sufficient statistical confidence. This operation allows for the harmonization of different modality-specific latent spaces and, thereby facilitates the learning of a joint score network.

### Appendix A.2. Multimodal Diffusion SDE

In Section 3, we presented our multimodal latent diffusion process allowing multimodal joint and conditional generation. The role of the SDE is to gradually add noise to the data, perturbing its structure until attaining a noise distribution. In this work, we consider Variance preserving SDE (VPSDE) [32]. In this framework, we have ρ(r)∼N(0;I), $\alpha \left(t\right)=-\frac{1}{2}\beta \left(t\right)$ and $g\left(t\right)=\sqrt{\beta (t)}$, where $\beta \left(t\right)=\beta_{min}+t\left(\beta_{max}-\beta_{min}\right)$. Following [32,59], we set $\beta_{min}=0.1$ and $\beta_{max}=20$. With this configuration, and by substitution of Equation (4), we obtain the following forward SDE:

(A1) $$dR_{t}=-\frac{1}{2}\beta \left(t\right)R_{t}dt+\sqrt{\beta (t)}dW_{t},\;t\in \left[0,T\right].$$

The corresponding perturbation kernel is provided by

(A2) $$q\left(r|z,t\right)=N(r;e^{-\frac{1}{4}t^{2}\left(\beta_{max}-\beta_{min}\right)-\frac{1}{2}t\beta_{min}}z,\left(1-e^{-\frac{1}{2}t^{2}\left(\beta_{max}-\beta_{min}\right)-t\beta_{min}}\right)I).$$

The marginal score $\nabla \log q(R_{t},t)$ is approximated by a score network $s_{\chi }\left(R_{t},t\right)$, the parameters χ of which can be optimized by minimizing the ELBO in Equation (6), where we found that using the same re-scaling as in [32] is more stable.

The reverse process is described by a different SDE (Equation (5)). When using a variance-preserving SDE, Equation (5) specializes in

(A3) $$dR_{t}=\left[\frac{1}{2}\beta \left(T-t\right)R_{t}+\beta \left(T-t\right)\nabla \log q\left(R_{t},T-t\right)\right]dt+\sqrt{\beta (T-t)}dW_{t},$$

with $R_{0}∼\rho \left(r\right)$ as the initial condition and time *t* flowing from t=0 to t=T.

When the parametric score network has been optimized through the simulation of Equation (A3), sampling $R_{T}∼q_{\psi }\left(r\right)$ becomes possible, allowing **joint generation**. A numerical SDE solver can be used to sample $R_{T}$, which can then be fed to the modality-specific decoders to jointly sample a set of $\hat{X}={\left\{d_{\theta }^{i}\left(R_{T}^{i}\right)\right\}}_{i=0}^{M}$. As explained in Section 4.3, the use of the unconditional score network $s_{\chi }\left(R_{t},t\right)$ allows for **conditional generation** through the approximation described in [32].

As described in Algorithm A1, we can generate a set of modalities $A_{1}$ conditioned on the available set of modalities $A_{2}$. The available modalities are encoded into their respective latent space $z^{A_{2}}$, the initial missing part is sampled from the stationary distribution $R_{0}^{A_{1}}∼\rho \left(r^{A_{1}}\right)$ using an SDE solver (e.g., Euler–Maruyama), and the reverse diffusion SDE in Equation (A3) is discretized using a finite time step Δt=T/N, starting from t=0 and iterating until t≈T. At each iteration, the available portion of the latent space is diffused and brought to the same noise level as $R_{t}^{A_{1}}$, allowing for the use of the unconditional score network. Lastly, the reverse diffusion update is performed. This process is repeated until arriving at t≈T and obtaining $R_{T}^{A_{1}}={\hat{Z}}^{A_{1}}$, which can be decoded to recover ${\hat{x}}^{A_{1}}$. Note that this joint generation can be seen as a special case of Algorithm A1 with $A_{2}=\emptyset$. We name this first approach Multi-modal Latent Diffusion with In-painting (MLD IN-PAINT), and provide extensive comparison with our MLD method in Appendix B.

**Algorithm A1:** MLD IN-PAINT conditional generation

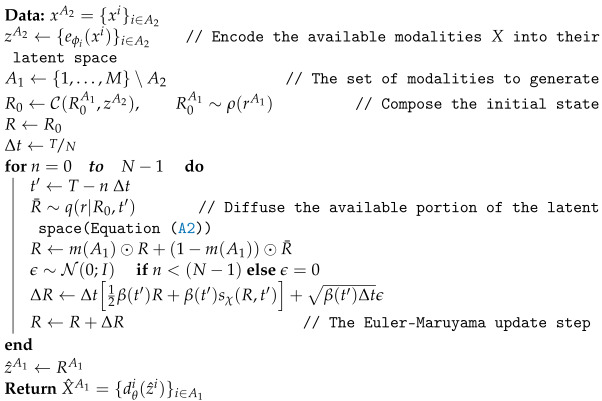

As discussed in Section 4.3, the approximation enabling the in-painting approach can be efficient in several domains; however, its generalization to the multimodal latent space scenario is not trivial. We argue that this is due to the heterogeneity of modalities. which induce different characteristics on the part of the latent spaces. For different modality-specific latent spaces, the loss of information ratio can vary through the diffusion process. We verify this hypothesis by the following experiment.

**Latent space robustness against diffusion perturbation.**

We analyse the effect of the forward diffusion perturbation on the latent space through time. We encode the modalities using their respective encoders to obtain their latent space $Z=[e_{\psi^{1}}\left(X^{1}\right)\ldots e_{\psi^{M}}\left(X^{M}\right)]$. Given a time t∈[0,T], we diffuse the different latent spaces by applying Equation (A2) to obtain $R_{t}∼q\left(r|z,t\right)$, with $R_{t}$ being the perturbed version of the latent space at time *t*. We feed the modality-specific decoders with the perturbed latent space ${\hat{X}}_{t}={\left\{d_{\theta }^{i}\left(R_{t}^{i}\right)\right\}}_{i=1}^{M}$, with ${\hat{X}}_{t}$ being the output modalities generated using the perturbed latent space. To evaluate the information loss induced by the diffusion process on the different modalities, we assess the coherence preservation in the reconstructed modalities ${\hat{X}}_{t}$ by computing the coherence (in %) as done in Section 5.

We expect to obtain high coherence results for t≈0 when compared to t≈T, asthe information in the latent space is more preserved at the beginning of the diffusion process than at the last phase of the forward SDE, where all dependencies on initial conditions vanish. Figure A1 shows the coherence as a function of the diffusion time t∈[0,1] for different modalities across multiple datasets. It can be observed that, within the same dataset, certain modalities stand out with a specific level of robustness (using the coherence level as a proxy) against the diffusion perturbation in comparison with the remaining modalities from the same dataset. For instance, we remark that SVHN is less robust than MNIST, which should manifest in underperformance of SVHN-to-MNIST conditional generation. We verify this intuition in Appendix B.

### Appendix A.3. Multi-Time Masked Multimodal SDE

In Section 4, we proposed a multi-time masked diffusion process to learn a score network capable of both conditional and joint generation.

Algorithm A2 presents the pseudo-code for the multi-time masked training. The masked diffusion process is applied following randomization with probability *d*. First, a subset of modalities $A_{2}$ is selected randomly to be the conditioning modalities, with $A_{1}$ the remaining set of modalities to make up the diffused modalities. The time *t* is sampled uniformly from [0,T], and the portion of the latent space corresponding to the subset $A_{1}$ is diffused accordingly. Using the masking as shown in Algorithm A2, the portion of the latent space corresponding to the subset $A_{2}$ is not diffused and is forced to be equal to $R_{0}^{A_{2}}=z^{A_{2}}$. The multi-time vector τ is constructed. Lastly, the score network is optimized by minimizing a masked loss corresponding to the diffused part of the latent space. With probability (1−d), all the modalities are diffused at the same time and $A_{2}=\emptyset$. In order to calibrate the loss, given that the randomization of $A_{1}$ and $A_{2}$ can result in diffusing different sizes of the latent space, we re-weight the loss according to the cardinality of the diffused and frozen portions of the latent space:

(A4) $$\Omega \left(A_{1},A_{2}\right)=1+\frac{dim(A_{2})}{dim(A_{1})},$$

where dim(.) is the sum of each latent space cardinality of a given subset of modalities with dim(∅)=0.

**Algorithm A2:** MLD masked multi-time diffusion training step
**Data**: $X={\left\{x^{i}\right\}}_{i=1}^{M}$
**Param:** *d*
$Z\leftarrow {\left\{e_{\phi_{i}}\left(x^{i}\right)\right\}}_{i=0}^{M}$ // Encode the modalities *X* into their latent space
$A_{2}∼\nu \;$ // ν depends on the parameter *d*
$A_{1}\leftarrow \left\{1,\ldots ,M\right\}\setminus A_{2}$
t∼U[0,T]
R∼q(r|Z,t) // Diffuse the available portion of the latent space (Equation (A2))
$R\leftarrow m\left(A_{1}\right)⊙R+\left(1-m\left(A_{1}\right)\right)⊙Z$ // Masked diffusion
$\tau \left(A_{1},t\right)\leftarrow \left[1\left(1\in A_{1}\right)t,\ldots ,1\left(M\in A_{1}\right)t\right]$ // Construct the multi time vector
**Return** $\nabla_{\chi }\left\{\Omega \left(A_{1},A_{2}\right)\;{\left\|m\left(A_{1}\right)⊙\;\left[s_{\chi }\left(R,\tau \left(A_{1},t\right)\right)-\nabla \log q\left(R,t|z^{A_{2}}\right)\right]\right\|}_{2}^{2}\;\right\}$

    The optimized score network can approximate both the conditional and unconditional true score:

(A5) $$s_{\chi }\left(R_{t},\tau \left(A_{1},t\right)\right)∼\nabla \log q\left(R_{t},t\;|\;z^{A_{2}}\right)).$$

Joint generation is a special case of the latter with $A_{2}=\emptyset$:

(A6) $$s_{\chi }\left(R_{t},\tau \left(A_{1},t\right)\right)∼\nabla \log q\left(R_{t},t\right)\;,A_{1}=\left\{1,\ldots ,M\right\}.$$

Algorithm A3 describes the reverse conditional generation pseudo-code. It is pertinent to compare this algorithm with Algorithm A1. The main difference resides in the use of the multi-time score network to enable conditional generation, with the multi-time vector playing the role of the time information and conditioning signal. On the other hand, in Algorithm A1, we do not have a conditional score network; therefore, we resort to the approximation from Section 4.3 and use the unconditional score.

**Algorithm A3:** MLD conditional generation.

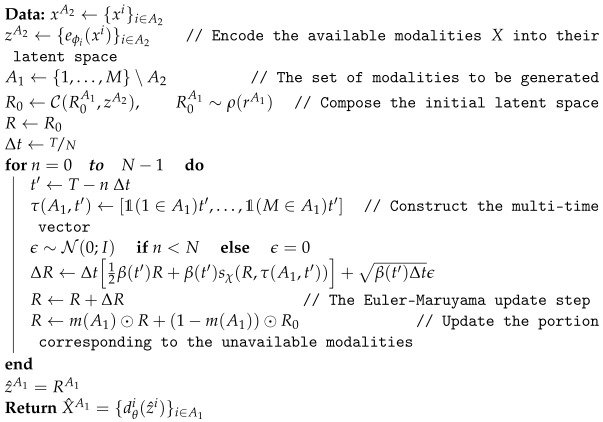

### Appendix A.4. Uni-Diffuser Training

The work presented in [35] is specialized for an image–caption application. The approach is based on a multimodal diffusion model applied to a unified latent embedding obtained via pretrained autoencoders and incorporates pretrained models (CLIP [60] and GPT-2 [61]). The unified latent space is composed of an image embedding, a CLIP image embedding, and a CLIP text embedding. Note that the CLIP model is pretrained on (image–text) pairs of multimodal data, which is expected to enhance the generative performance. Because it is non-trivial to have a jointly trained encoder similar to CLIP for any type of modality, the evaluation of this model on different modalities across different datasets (e.g., including audio) is not an easy task.

To compare to this work, we adapted the training scheme presented in [35] to our MLD method. Instead of applying a masked multimodal SDE to train the score network, every portion of the latent space was diffused according to a different time $t^{i}∼U\left(0,1\right)$; therefore, the multi-time vector fed to the score network was $\tau \left(t\right)=[t^{0}∼U\left(0,1\right),\ldots ,t^{M}∼U\left(0,1\right)]$. For fairness, we used the same score network and reverse process sampler as was used for our MLD version with multi-time training; we call this variant Multi-modal Latent Diffusion UniDiffuser (MLD UNI).

### Appendix A.5. Technical Details

#### Appendix A.5.1. Sampling Schedule

We used the sampling schedule proposed in [53], which has been shown to improve the coherence of conditional and joint generation. We used the best parameters suggested by the authors: N=250 time steps applied r=10 resampling times with jump size j=10. For readability, in Algorithms A1 and A3 we present pseudo-code with a linear sampling schedule which can be easily adapted to any other schedule.

#### Appendix A.5.2. Training the Score Network

Inspired by the architecture from [62], we use simple Residual MLP blocks with skip connections as our score network (see Figure A2). We fix the **width** and **number of blocks** proportionally to the number of the modalities and the latent space size. As in [63], we use the Exponential moving average (EMA) of the model parameters with a momentum parameter m=0.999.

## Appendix B. MLD Ablation Study

In this section, we compare MLD with two variants presented in Appendix A: MLD IN-PAINT, a naive approach without our proposed *multi-time masked* SDE, and MLD UNI, a variant of our method using the same training scheme from [35]. In addition, we analyze the effect of the randomization parameter *d* on the performance of MLD through an ablation study.

### Appendix B.1. MLD and Its Variants

Table A1 summarizes the different approaches adopted in each variant. All the considered models share the same deterministic autoencoders trained during the first stage.

For fairness, our evaluation was carried out using the same configuration and code basis as MLD. This included the autoencoder architectures and latent space size (similar to Section 5). The same score network (Figure A2) was used across experiments, with MLD IN-PAINT using the same architecture with one time dimension instead of the multi-time vector. In all the variants, joint and conditional generation were conducted using the same reverse sampling schedule described in Appendix A.

**Table A1:** Ablation study of MLD and its variants.

Model	Multi-Time Diffusion	Training	Conditional and Joint Generation
MLD IN-PAINT	✕	Equation (6)	Algorithm A1
MLD UNI	✓	[35]	Algorithm A3
MLD	✓	Algorithm A2	Algorithm A3

#### Appendix B.1.1. Results

In certain cases, the MLD variants were able to match the joint generation performance of MLD; however, overall they were less efficient and had noticeable weaknesses. MLD IN-PAINT underperforms on conditional generation when considering relatively complex modalities, while MLD UNI is not able to leverage the presence of multiple modalities to improve cross-generation, especially for datasets with a large number of modalities. On the other hand, MLD is able to overcome these limitations.

#### Appendix B.1.2. MNIST-SVHN

In Table A2, MLD achieves the best results and dominates cross=generation performance. It can be observed that MLD IN-PAINT lacks coherence for SVHN-to-MNIST conditional generation, a result we expected based on our analysis of the experiment in Figure A1. MLD UNI, despite the use of a multi-time diffusion process, underperforms our method, which indicates the effectiveness of our masked diffusion process in learning the conditional score network. Because all of the models used the same deterministic autoencoders, their observed generative quality performances are relatively similar (see Figure A4 for qualitative results).

**Table A2:** Generation coherence and quality for MNIST-SVHN (M stands for MNIST and S for SVHN). The generation quality is measured in terms of FMD for MNIST and FID for SVHN. Bold and underlined numbers indicate the best and second best scores respectively.

Models	Coherence (%↑)			Quality (↓)			
	Joint	M→S	S→M	Joint (M)	Joint (S)	M→S	S→M
MLD-Inpaint	$85.53_{\pm 0.22}$	${\underline{81.76}}_{\pm 0.23}$	$63.28_{\pm 1.16}$	$3.85_{\pm 0.02}$	$60.86_{\pm 1.27}$	$59.86_{\pm 1.18}$	$3.55_{\pm 0.11}$
MLD-Uni	$82.19_{\pm 0.97}$	$79.31_{\pm 1.21}$	${\underline{72.78}}_{\pm 1.81}$	$4.1_{\pm 0.17}$	$57.41_{\pm 1.43}$	${\underline{57.84}}_{\pm 1.57}$	$4.84_{\pm 0.28}$
MLD	${\underline{85.22}}_{\pm 0.5}$	$83.79_{\pm 0.62}$	$79.13_{\pm 0.38}$	${\underline{3.93}}_{\pm 0.12}$	$56.36_{\pm 1.63}$	$57.2_{\pm 1.47}$	${\underline{3.67}}_{\pm 0.14}$

#### Appendix B.1.3. MHD

Table A3 shows the performance results for the MHD dataset in terms of generative coherence. MLD achieves the best joint generation coherence, and, dominates the cross-generation coherence results along with MLD UNI. MLD IN-PAINT shows a lack of coherence when conditioning on the sound modality alone, which is a predictable result, as this is a more difficult configuration because the sound modality is loosely correlated to other modalities. It can be observed that MLD IN-PAINT performs worse than the two other alternatives when conditioned on the trajectory modality, which is the smallest modality in terms of latent size. This indicates another limitation of the naive approach regarding coherent generation when handling different latent spaces sizes, a weakness that our MLD method overcomes. Table A4 presents the qualitative generative performance results, which are homogeneous across the variants, with MLD achieving either the best or second-best performance.

**Table A3:** Generation coherence (%↑) for MHD (higher is better). The line above refers to the generated modality, while the subset of observed modalities is presented below. Bold and underlined numbers indicate the best and second best scores respectively.

Models	Joint	I (Image)			T (Trajectory)			S (Sound)		
		T	S	T,S	I	S	I,S	I	T	I,T
MLD-Inpaint	$96.88_{\pm 0.35}$	$63.9_{\pm 1.7}$	$56.52_{\pm 1.89}$	$95.83_{\pm 0.48}$	${\underline{99.58}}_{\pm 0.1}$	$56.51_{\pm 1.89}$	${\underline{99.89}}_{\pm 0.04}$	$95.81_{\pm 0.25}$	$56.51_{\pm 1.89}$	$96.38_{\pm 0.35}$
MLD-Uni	${\underline{97.69}}_{\pm 0.26}$	$99.91_{\pm 0.04}$	$89.87_{\pm 0.38}$	$99.92_{\pm 0.04}$	$99.68_{\pm 0.1}$	$89.78_{\pm 0.45}$	$99.38_{\pm 0.31}$	${\underline{97.54}}_{\pm 0.2}$	${\underline{97.65}}_{\pm 0.41}$	${\underline{97.79}}_{\pm 0.41}$
MLD	$98.34_{\pm 0.22}$	$99.45_{\pm 0.09}$	${\underline{88.91}}_{\pm 0.54}$	${\underline{99.88}}_{\pm 0.04}$	${\underline{99.58}}_{\pm 0.03}$	${\underline{88.92}}_{\pm 0.53}$	$99.91_{\pm 0.02}$	$97.63_{\pm 0.14}$	$97.7_{\pm 0.34}$	$98.01_{\pm 0.21}$

**Table A4:** Generation quality for MHD. The metrics reported are FMD for the image and trajectory modalities and FAD for the sound modality (lower is better). Bold and underlined numbers indicate the best and second best scores respectively.

Models	I (Image)				T (Trajectory)				S (Sound)			
	Joint	T	S	T,S	Joint	I	S	I,S	Joint	I	T	I,T
MLD-Inpaint	$5.35_{\pm 1.35}$	$6.23_{\pm 1.13}$	${\underline{4.76}}_{\pm 0.68}$	$3.53_{\pm 0.36}$	$1.59_{\pm 0.12}$	$0.6_{\pm 0.05}$	$1.81_{\pm 0.13}$	$0.54_{\pm 0.06}$	$2.41_{\pm 0.07}$	$2.5_{\pm 0.04}$	$2.52_{\pm 0.02}$	$2.49_{\pm 0.05}$
MLD-Uni	$7.91_{\pm 2.2}$	$1.65_{\pm 0.33}$	$6.29_{\pm 1.38}$	${\underline{3.06}}_{\pm 0.54}$	${\underline{2.53}}_{\pm 0.5}$	$1.18_{\pm 0.26}$	$3.18_{\pm 0.77}$	$2.84_{\pm 1.14}$	$2.11_{\pm 0.08}$	$2.25_{\pm 0.05}$	$2.1_{\pm 0.0}$	$2.15_{\pm 0.01}$
MLD	${\underline{7.98}}_{\pm 1.41}$	${\underline{1.7}}_{\pm 0.14}$	$4.54_{\pm 0.45}$	$1.84_{\pm 0.27}$	$3.18_{\pm 0.18}$	${\underline{0.83}}_{\pm 0.03}$	${\underline{2.07}}_{\pm 0.26}$	${\underline{0.6}}_{\pm 0.05}$	${\underline{2.39}}_{\pm 0.1}$	${\underline{2.31}}_{\pm 0.07}$	${\underline{2.33}}_{\pm 0.11}$	${\underline{2.29}}_{\pm 0.06}$

#### Appendix B.1.4. POLYMNIST

In Figure A5, we note the superiority of MLD in both generative coherence and quality. MLD-Uni is not able to leverage the presence of a large number of modalities in conditional generation coherence. Interestingly, an increase in the number of input modalities negatively impacts the performance of MLD UNI.

#### Appendix B.1.5. CUB

Figure A6 shows the qualitative results for caption-to-image conditional generation. All of the variants are based on the same first-stage autoencoders, and the generative performance is comparable in terms of quality.

### Appendix B.2. Randomization d-Ablation Study

The *d* parameter controls the randomization of the *multi-time masked diffusion process* during training in Algorithm A2. With probability *d*, the concatenated latent space corresponding to all the modalities is diffused at the same time. With probability (1−d), a portion of the latent space corresponding to a random subset of the modalities is not diffused and is frozen during the training step. To study the *d* parameter and its effect on the performance of our MLD model, we used d∈{0.1,*…*, 0.9}. Figure A7 shows the results of the *d*-ablation study on the **MNIST-SVHN** dataset. We report the performance results averaged over five independent seeds as a function of the probability (1−d): **Left** shows the conditional and joint coherence for the **MNIST-SVHN** dataset; **Middle** shows the quality performance in terms of FID for SVHN generation; and **Right** shows the quality performance in terms of FMD for MNIST generation.

It can be observed that higher values for 1−d, indicating a greater probability of applying *multi-time masked diffusion*, improve the coherence of SVHN-to-MNIST conditional generation. This confirms that masked multi-time training enables better conditional generation. Overall, on the **MNIST-SVHN** dataset, MLD shows weak sensibility to the *d* parameter whenever the value of d∈[0.2,0.7].

## Appendix C. Datasets and Evaluation Protocol

### Appendix C.1. Dataset Description

**MNIST-SVHN** [22] is constructed using pairs of MNIST and SVHN sharing the same digit class (see Figure A8a). Each instance of a digit class (in either dataset) is randomly paired with 20 instances of the same digit class from the other dataset. SVHN modality samples are obtained from house numbers in Google Street View images, and are characterized by a variety of colors, shapes, and angles. A high number of SVHN samples are noisy, and can contain different digits within the same sample due to the imperfect cropping of the original full house number image. One challenge of this dataset for multimodal generative models is to learn to extract digit number and reconstruct a coherent MNIST modality.

**MHD** [26] is composed of three modalities: synthetically generated images and motion trajectories of handwritten digits associated with their speech sounds. The images are gray-scale 1 × 28 × 28, and the handwriting trajectories are represented by a 1 × 200 vector. The spoken digits sounds are 1s audio clips processed as Mel-Spectrograms, and are constructed with a hopping window of 512 ms with 128 Mel Bins, resulting in a 1 × 128 × 32 representation. This benchmark is the closest to a real-world scenario involving multimodal sensors because of the presence of three completely different modalities, with the audio modality representing a complex data type. Therefore, similar to SVHN, the conditional generation of sound to coherent images or trajectories represents a challenging use case.

**POLYMNIST** [23] is a version of the MNIST dataset extended to five modalities. Each modality is constructed using a random set of MNIST digits with an overlay over a random crop from a modality-specific three-channel image background. This synthetic generated dataset allows for the evaluating the scalability of multimodal generative models to large number of modalities. Although this dataset is composed only of images, the different textures of different modality-specific backgrounds results in differing levels of difficulty. In Figure A8c, the digits are more difficult to distinguish in modalities 1 and 5 than in the other modalities.

**CUB** [22] is comprised of bird images and associated text captions. In [22], a simplified version based on precomputed ResNet-features was used. Following [24], we conducted all of our experiments on the real image data instead. Each image from the 11,788 photos of birds from Caltech-Birds [64] was resized to a 3 × 64 × 64 image and coupled with ten textual descriptions of the respective bird (see Figure A8d).

**CelebAHQ-mask** consists of three modalities: face images, each with a segmentation mask and attributes. We took into account 18 out of 40 attributes from the original dataset and resized the images to 128 × 128 resolution, as was done in [21,36].

### Appendix C.2. Evaluation Metrics

The multimodal generative models were evaluated in terms of their generative coherence and quality.

#### Appendix C.2.1. Generation Coherence

We measured *coherence* by verifying that generated data for both joint and conditional generation shared the same information across modalities. Following [22,23,24,26,27], we considered the class label of the modalities as the shared information and used pretrained classifiers to extract the label information from the generated samples and compare it across modalities.

For **MNIST-SVHN**, **MHD**, and **POLYMNIST**, the shared semantic information is the digit class number. Single-modality classifiers are trained to classify the digit number of a given modality sample. To compute the conditional generation of a modality *m* with a subset of modalities *A*, the conditional generated sample ${\hat{X}}^{m}$ is fed to the modality-specific pretrained classifier $C_{m}$. The predicted label class is compared to the ground truth label $y_{X^{A}}$, which is the label of the modalities in subset $X^{A}$. For *N* samples, the matching rate average establishes the coherence. For all the experiments, *N* was equal to the length of the test set.

(A7) $$\mathrm{Coherence}\left({\hat{X}}^{m}|X^{A}\right)=\frac{1}{N}\sum_{1}^{N}1_{\left\{C_{m}\left({\hat{X}}^{m}\right)=y_{X^{A}}\right\}}$$

The **joint generation coherence** was measured by feeding the generated samples of each modality to their specific trained classifier. The rate at which all classifiers output the same predicted digit label for *N* generations was considered the joint generation coherence.

**The leave-one-out coherence** is the conditional generation coherence using all possible subsets excluding the generated modality ($\mathrm{Coherence}({\hat{X}}^{m}|X^{A})$ with A={1,..,M}∖m). Due to the large number of modalities in **POLYMNIST**, similar to [23,24,27], we computed the average **leave-one-out coherence** conditional coherence as a function of the subset size of the input modalities.

Due to the unavailability of labels in the **CUB** dataset, we used CLIP-S [56], a state-of-the art metric for image captioning evaluation.

#### Appendix C.2.2. Generation Quality

For each modality, we considered the following metrics:

- **RGB Images**: FID [54] is the state-of-the-art standard metric for evaluating the image generation quality of generative models.
- **Audio**: FAD [55] is a state-of-the-art standard metric for the evaluation of audio generation. FAD performs well in terms of robustness against noise, and is consistent with human judgments [65]. Similar to FID, the Fréchet distance is computed, except that VGGish (audio classifer model) embeddings are used instead.
- **Other modalities**: For other modality types, we derived the FMD (Fréchet Modality Distance), a similar metric to FID and FAD. We computed the **Fréchet distance** between the statistics retrieved from the activations of the modality-specific pretrained classifiers used for coherence evaluation. FMD was used to evaluate the generative quality of the MNIST modality on the **MNIST-SVHN** dataset and the image and trajectory modalities on the **MHD** dataset.

For conditional generation, we computed the quality metric (FID, FAD, or FMD) using the conditionally generated modality and the real data. For joint generation, we used the randomly generated modality and randomly selected the same number of samples from the real data.

For **CUB**, we used 10,000 samples to evaluate the generation quality in terms of FID. In the remaining experiments, we used 5000 samples to evaluate the performance in terms of FID, FAD, or FMD.

## Appendix D. Implementation Details

In this section, we report the implementation details for each benchmark. We used the same unified code base for all the baselines, relying on the *PyTorch* framework. The VAE implementation was adapted from the official code whenever available (MVAE, MMVAE and MOPOE, as in (https://github.com/thomassutter/MoPoE (accessed on 11 February 2024)), MVTCAE (https://github.com/gr8joo/MVTCAE (accessed on 11 February 2024)), and NEXUS (https://github.com/miguelsvasco/nexus_pytorch (accessed on 11 February 2024)). To ensure fairness, MLD and all VAE-based models used the same autoencoder architecture. We used the best hyperparameters suggested by the authors. Across all the datasets, we used the *Adam optimizer* [66] for training.

### Appendix D.1. MLD

MLD used the same autoencoder architecture as for VAE-based models, except that the latter are deterministic autoencoders. The autoencoders were trained using the same reconstruction loss term as for the VAE-based models. Table A5 and Table A6 summarize the hyperparameters used during the two phases of MLD training. Note that data augmentation was necessary for the image modality in the CUB dataset in order to overcome overfitting when training the deterministic autoencoder. For this, we used *TrivialAugmentWide* from the Torchvision library.

**Table A5:** MLD: hyperparameters used for the deterministic autoencoders.

Dataset	Modality	Latent Space	Batch Size	Lr	Epochs	Weight Decay
**MNIST-SVHN**	MNIST	16	128	$1\times 10^{-3}$	150	
	SVHN	64				
**MHD**	Image	64	64	$1\times 10^{-3}$	500	
	Trajectory	16				
	Sound	128				
**POLYMNIST**	All modalities	160	128	$1\times 10^{-3}$	300	
**CUB**	Caption	32	128	$1\times 10^{-3}$	500	
	Image	64		$1\times 10^{-4}$	300	$1\times 10^{-6}$
**CelebAMask-HQ**	Image	256	64	$1\times 10^{-3}$	200	
	Mask	128				
	Attributes	32				

**Table A6:** MLD: score network hyperparameters.

Dataset	*d*	Blocks	Width	Time Embed	Batch Size	Lr	Epochs
**MNIST-SVHN**	0.5	2	512	256	128		150
**MHD**	0.3	2	1024	512	128		3000
**POLYMNIST**	0.5	2	1536	512	256	$1\times 10^{-4}$	3000
**CUB**	0.7	2	1024	512	64		3000
**CelebAMask-HQ**	0.5	2	1536	512	64		3000

### Appendix D.2. VAE-Based Models

For **MNIST-SVHN**, we followed [22,23] and used the same autoencoder architecture and pretrained classifier. The latent space size was set to 20, β=5.0. For MVTCAE $\alpha =\frac{5}{6}$. For both modalities, the likelihood was estimated using the Laplace distribution. For NEXUS, we used the same modality latent space size as in MLD, the joint NEXUS latent space was set to 20, $\beta_{i}=1.0$, and $\beta_{c}=5.0$. We trained all the VAE-models for 150 epochs with a batch size of 256 and learning rate of $1\times 10^{-3}$.

For **MHD**, we reused the autoencoder architecture and pretrained classifier from [26]. We adopted the hyperparameters from [26] to train the NEXUS model with the same settings while discarding the label modality. For the remaining VAE-based models, the latent space size was set to 128, β=1.0, and $\alpha =\frac{5}{6}$ for MVTCAE. For all the modalities, Mean square error (MSE) was used to compute the reconstruction loss, similar to [26]. The models were trained for 600 epochs with a batch size of 128 and learning rate of $1\times 10^{-3}$.

For **POLYMNIST**, we used the same autoencoder architecture and pretrained classifier used by [23,27]. We set the latent space size to 512, β=2.5, and $\alpha =\frac{5}{6}$ for MVTCAE. For all the modalities, the likelihood was estimated using the Laplace distribution. For NEXUS, we used the same modality latent space size as in MLD, the joint NEXUS latent space was 64, $\beta_{i}=1.0$, and $\beta_{c}=2.5$. We trained all the models for 300 epochs with a batch size of 256 and learning rate of $1\times 10^{-3}$.

For **CUB**, we used the same autoencoder architecture and implementation settings as in [24]. The Laplace and one-hot categorical distributions were used to estimate the likelihoods of the image and caption modalities, respectively. The latent space size was set to 64, β=9.0 for MVAE, MVTCAE, and MOPOE, and β=1 for MMVAE. We set $\alpha =\frac{5}{6}$ for MVTCAE. For NEXUS, we used the same modality latent space sizes as in MLD, the joint NEXUS latent space was set to 64, $\beta_{i}=1.0$, and $\beta_{c}=1$. We trained all the models for 150 epochs with a batch size of 64. We used a learning rate of 5e−4 for MVAE, MVTCAE, and MOPOE and $1\times 10^{-3}$ for the remaining models.

Finally, we note that in the official implementation of [23,27] on the **POLYMNIST** and **MNIST-SVHN** datasets, the classifiers were used for evaluation with dropout. In our implementation, we made sure to deactivate dropout during the evaluation step.

For **CelebAMask-HQ**, in our MLD experiments we used deterministic autoencoders instead of variational autoencoders [58].

### Appendix D.3. MLD with Powerful Autoencoder

Here, we provide more detail about the CUB experiment using a more powerful autoencoder, denoted MLD* in Figure 5. We used an architecture similar to [10] adapted to (64 × 64) resolution images. We modified the autoencoder architecture to be deterministic and trained the model with a simple mean square error loss. We kept the same configuration as the CUB experiment described in the previous experiment on the same dataset, including the text autoencoder, score network, and hyperparameters. We performed further experiments with the same settings on 128 × 128 resolution images. We include the qualitative results in Figure A21.

### Appendix D.4. Computation Resources

In our experiments, we used four A100 GPUs for a total of roughly four months of experiments.

## Appendix E. Additional Results

In this section, we report detailed results for all of our experiments, including the standard deviation and additional qualitative samples for all the datasets and all the methods we compared in our work.

### Appendix E.1. MNIST-SVHN

#### Appendix E.1.1. Self-Reconstruction

In Table A7, we report the results on *self-coherence*, which we use to support the arguments from Section 2. This metric is used to measure the loss of information due to latent collapse by showing the ability of all competing models to reconstruct an arbitrary modality given the same modality or a set thereof as an input. For our MLD model, self-reconstruction is done without using the diffusion model component; the modality is encoded using its deterministic encoder, and the decoder is fed the latent space to obtain the reconstruction.

We observe that the VAE-based models fail to reconstruct SVHN given SVHN. This is especially visible for the models based on the product-of-experts approach (MVAE and MVTCAE). In MLD, the deterministic autoencoders do not suffer from such weakness, and achieve the best overall performance.

Figure A9 shows the qualitative self-generation results. We remark that the digits in certain samples generated using VAE-based models differ from those in the input sample (for example, generation of the MNIST digit 3 in the case of MVAE and the SVHN digit 2 in the case of MVTCAE), indicating information loss due to latent collapse.

**Table A7:** Self-generation coherence and quality for **MNIST-SVHN** (M: MNIST, S: SVHN). The generation quality is measured in terms of FMD for MNIST and FID for SVHN. Bold and underlined numbers indicate the best and second best scores respectively.

Models	Coherence (%↑)				Quality (↓)			
	M→M	M,S→M	S→S	M,S→S	M→M	M,S→M	S→S	M,S→S
MVAE	$86.92_{\pm 0.8}$	$88.03_{\pm 0.78}$	$40.62_{\pm 0.99}$	$68.01_{\pm 1.29}$	$10.75_{\pm 1.04}$	$10.79_{\pm 1.02}$	$60.22_{\pm 1.01}$	$59.0_{\pm 0.6}$
MMVAE	$87.22_{\pm 1.87}$	$77.35_{\pm 4.19}$	$67.31_{\pm 6.93}$	$39.44_{\pm 3.43}$	$12.15_{\pm 1.25}$	$20.24_{\pm 1.04}$	$58.1_{\pm 3.14}$	$171.42_{\pm 4.55}$
MOPOE	$89.95_{\pm 0.84}$	$91.71_{\pm 0.77}$	$67.26_{\pm 0.8}$	${\underline{83.58}}_{\pm 0.44}$	$9.39_{\pm 0.76}$	$10.1_{\pm 0.73}$	$53.19_{\pm 1.06}$	$57.34_{\pm 1.35}$
NEXUS	$92.63_{\pm 0.45}$	$93.59_{\pm 0.4}$	${\underline{68.31}}_{\pm 0.46}$	$83.13_{\pm 0.58}$	$4.92_{\pm 0.61}$	$5.16_{\pm 0.59}$	$85.67_{\pm 2.74}$	$97.86_{\pm 2.86}$
MVTCAE	${\underline{94.33}}_{\pm 0.18}$	${\underline{95.18}}_{\pm 0.19}$	$47.47_{\pm 0.76}$	$86.6_{\pm 0.23}$	${\underline{4.67}}_{\pm 0.35}$	${\underline{4.94}}_{\pm 0.37}$	${\underline{52.29}}_{\pm 1.17}$	${\underline{53.55}}_{\pm 1.19}$
MLD	$96.73_{\pm 0.0}$	96.73±0.0	$82.19_{\pm 0.0}$	$82.19_{\pm 0.0}$	$2.25_{\pm 0.03}$	2.25±0.03	$48.47_{\pm 0.63}$	$48.47_{\pm 0.63}$

#### Appendix E.1.2. Detailed Results

**Table A8:** Generative coherence for **MNIST-SVHN**. We report the detailed version of Table 1 with the standard deviation for five independent runs with different seeds. Bold and underlined numbers indicate the best and second best scores respectively.

Models	Coherence (%↑)			Quality (↓)			
	Joint	M→S	S→M	Joint(M)	Joint(S)	M → S	S→M
MVAE	$38.19_{\pm 2.27}$	$48.21_{\pm 2.56}$	$28.57_{\pm 1.46}$	$13.34_{\pm 0.93}$	$68.0_{\pm 0.99}$	$68.9_{\pm 1.84}$	$13.66_{\pm 0.95}$
MMVAE	$37.82_{\pm 1.19}$	$11.72_{\pm 0.33}$	$67.55_{\pm 9.22}$	$25.89_{\pm 0.46}$	$146.82_{\pm 4.76}$	$393.33_{\pm 4.86}$	$53.37_{\pm 1.87}$
MOPOE	$39.93_{\pm 1.54}$	$12.27_{\pm 0.68}$	$68.82_{\pm 0.39}$	$20.11_{\pm 0.96}$	$129.2_{\pm 6.33}$	$373.73_{\pm 26.42}$	$43.34_{\pm 1.72}$
NEXUS	$40.0_{\pm 2.74}$	$16.68_{\pm 5.93}$	$70.67_{\pm 0.77}$	$13.84_{\pm 1.41}$	$98.13_{\pm 5.9}$	$281.28_{\pm 16.07}$	$53.41_{\pm 1.54}$
MVTCAE	$48.78_{\pm 1}$	${\underline{81.97}}_{\pm 0.32}$	$49.78_{\pm 0.88}$	$12.98_{\pm 0.68}$	$52.92_{\pm 1.39}$	$69.48_{\pm 1.64}$	$13.55_{\pm 0.8}$
MMVAE+	$17.64_{\pm 4.12}$	$13.23_{\pm 4.96}$	$29.69_{\pm 5.08}$	$26.60_{\pm 2.58}$	$121.77_{\pm 37.77}$	$240.90_{\pm 85.74}$	$35.11_{\pm 4.25}$
MMVAE+ (K = 10)	$41.59_{\pm 4.89}$	$55.3_{\pm 9.89}$	$56.41_{\pm 5.37}$	$19.05_{\pm 1.10}$	$67.13_{\pm 4.58}$	$75.9_{\pm 12.91}$	$18.16_{\pm 2.20}$
MLD	${\underline{85.22}}_{\pm 0.5}$	$83.79_{\pm 0.62}$	$79.13_{\pm 0.38}$	${\underline{3.93}}_{\pm 0.12}$	${\underline{56.36}}_{\pm 1.63}$	$57.2_{\pm 1.47}$	${\underline{3.67}}_{\pm 0.14}$

### Appendix E.2. MHD

**Table A9:** Generative coherence for **MHD**. We report the detailed version of Table 2 with the standard deviation for five independent runs with different seeds. Bold and underlined numbers indicate the best and second best scores respectively.

Models	Joint	I (Image)			T (Trajectory)			S (Sound)		
		T	S	T,S	I	S	I,S	I	T	I,T
MVAE	$37.77_{\pm 3.32}$	$11.68_{\pm 0.35}$	$26.46_{\pm 1.84}$	$28.4_{\pm 1.47}$	$95.55_{\pm 1.39}$	$26.66_{\pm 1.72}$	$96.58_{\pm 1.06}$	$58.87_{\pm 4.89}$	$10.39_{\pm 0.42}$	$58.16_{\pm 5.24}$
MMVAE	$34.78_{\pm 0.83}$	$99.7_{\pm 0.03}$	$69.69_{\pm 1.66}$	$84.74_{\pm 0.95}$	${\underline{99.3}}_{\pm 0.07}$	$85.46_{\pm 1.57}$	$92.39_{\pm 0.95}$	$49.95_{\pm 0.79}$	$50.14_{\pm 0.89}$	$50.17_{\pm 0.99}$
MOPOE	$48.84_{\pm 0.36}$	${\underline{99.64}}_{\pm 0.08}$	$68.67_{\pm 2.07}$	${\underline{99.69}}_{\pm 0.04}$	$99.28_{\pm 0.08}$	${\underline{87.42}}_{\pm 0.41}$	$99.35_{\pm 0.04}$	$50.73_{\pm 3.72}$	$51.5_{\pm 3.52}$	$56.97_{\pm 6.34}$
NEXUS	$26.56_{\pm 1.71}$	$94.58_{\pm 0.34}$	${\underline{83.1}}_{\pm 0.74}$	$95.27_{\pm 0.52}$	$88.51_{\pm 0.64}$	$76.82_{\pm 3.63}$	$93.27_{\pm 0.91}$	$70.06_{\pm 2.83}$	$75.84_{\pm 2.53}$	$89.48_{\pm 3.24}$
MVTCAE	$42.28_{\pm 1.12}$	$99.54_{\pm 0.07}$	$72.05_{\pm 0.95}$	$99.63_{\pm 0.05}$	$99.22_{\pm 0.08}$	$72.03_{\pm 0.48}$	${\underline{99.39}}_{\pm 0.02}$	${\underline{92.58}}_{\pm 0.47}$	${\underline{93.07}}_{\pm 0.36}$	${\underline{94.78}}_{\pm 0.25}$
MMVAE+	$41.67_{\pm 2.3}$	$98.05_{\pm 0.19}$	$84.16_{\pm 0.57}$	$91.88_{\pm }$	$97.47_{\pm 0.89}$	$81.16_{\pm 2.24}$	$89.31_{\pm 1.54}$	$64.34_{\pm 4.46}$	$65.42_{\pm 5.42}$	$64.88_{\pm 4.93}$
MMVAE+ (K = 10)	$42.60_{\pm 2.5}$	$99.44_{\pm 0.07}$	$89.75_{\pm 0.75}$	$94.7_{\pm 0.72}$	$99.44_{\pm 0.18}$	$89.58_{\pm 0.4}$	$95.01_{\pm 0.30}$	$87.15_{\pm 2.81}$	$87.99_{\pm 2.55}$	$87.57_{\pm 2.09}$
MLD	$98.34_{\pm 0.22}$	$99.45_{\pm 0.09}$	${\underline{88.91}}_{\pm 0.54}$	$99.88_{\pm 0.04}$	$99.58_{\pm 0.03}$	${\underline{88.92}}_{\pm 0.53}$	$99.91_{\pm 0.02}$	$97.63_{\pm 0.14}$	$97.7_{\pm 0.34}$	$98.01_{\pm 0.21}$

**Table A10:** Generative quality for **MHD**. We report the detailed version of Table 3 with the standard deviation for five independent runs with different seeds. Bold and underlined numbers indicate the best and second best scores respectively.

Models	I (Image)				T (Trajectory)				S (Sound)			
	Joint	T	S	T,S	Joint	I	S	I,S	Joint	I	T	I,T
MVAE	${\underline{94.9}}_{\pm 7.37}$	$93.73_{\pm 5.44}$	$92.55_{\pm 7.37}$	$91.08_{\pm 10.24}$	$39.51_{\pm 6.04}$	$20.42_{\pm 4.42}$	$38.77_{\pm 6.29}$	$19.25_{\pm 4.26}$	$14.14_{\pm 0.25}$	${\underline{14.13}}_{\pm 0.19}$	$14.08_{\pm 0.24}$	$14.17_{\pm 4.26}$
MMVAE	$224.01_{\pm 12.58}$	$22.6_{\pm 4.3}$	$789.12_{\pm 12.58}$	$170.41_{\pm 8.06}$	$16.52_{\pm 1.17}$	$0.5_{\pm 0.05}$	$30.39_{\pm 1.38}$	$6.07_{\pm 0.37}$	$22.8_{\pm 0.39}$	$22.61_{\pm 0.75}$	$23.72_{\pm 0.86}$	$23.01_{\pm 0.67}$
MOPOE	$147.81_{\pm 10.37}$	$16.29_{\pm 0.85}$	$838.38_{\pm 10.84}$	$15.89_{\pm 1.96}$	${\underline{13.92}}_{\pm 0.96}$	${\underline{0.52}}_{\pm 0.12}$	$33.38_{\pm 1.14}$	$0.53_{\pm 0.1}$	$18.53_{\pm 0.27}$	$24.11_{\pm 0.4}$	$24.1_{\pm 0.41}$	$23.93_{\pm 0.87}$
NEXUS	$281.76_{\pm 12.69}$	$116.65_{\pm 9.99}$	$282.34_{\pm 12.69}$	$117.24_{\pm 8.53}$	$18.59_{\pm 2.16}$	$6.67_{\pm 0.23}$	$33.01_{\pm 3.41}$	$7.54_{\pm 0.29}$	${\underline{13.99}}_{\pm 0.9}$	$19.52_{\pm 0.14}$	$18.71_{\pm 0.24}$	$16.3_{\pm 0.59}$
MVTCAE	$121.85_{\pm 3.44}$	${\underline{5.34}}_{\pm 0.33}$	${\underline{54.57}}_{\pm 7.79}$	${\underline{3.16}}_{\pm 0.26}$	$19.49_{\pm 0.67}$	$0.62_{\pm 0.1}$	${\underline{13.65}}_{\pm 1.24}$	$0.75_{\pm 0.13}$	$15.88_{\pm 0.19}$	$14.22_{\pm 0.27}$	${\underline{14.02}}_{\pm 0.14}$	${\underline{13.96}}_{\pm 0.28}$
MMVAE+	$97.19_{\pm 12.37}$	$2.80_{\pm 0.42}$	$128.56_{\pm 4.47}$	$114.3_{\pm 11.4}$	$22.37_{\pm 1.87}$	$1.21_{\pm 0.22}$	$21.74_{\pm 3.49}$	$15.2_{\pm 1.15}$	$16.12_{\pm 0.40}$	$17.31_{\pm 0.62}$	$17.92_{\pm 0.19}$	$17.56_{\pm 0.48}$
MMVAE+ (K = 10)	$85.98_{\pm 1.25}$	$1.83_{\pm 0.26}$	$70.72_{\pm 1.76}$	$62.43_{\pm 3.4}$	$21.10_{\pm 1.25}$	$1.38_{\pm 0.34}$	$8.52_{\pm 0.79}$	$7.22_{\pm 1.6}$	$14.58_{\pm 0.47}$	$14.33_{\pm 0.51}$	$14.34_{\pm 0.42}$	$14.32_{\pm 0.6}$
**MLD (ours)**	$7.98_{\pm 1.41}$	$1.7_{\pm 0.14}$	$4.54_{\pm 0.45}$	$1.84_{\pm 0.27}$	$3.18_{\pm 0.18}$	$0.83_{\pm 0.03}$	$2.07_{\pm 0.26}$	${\underline{0.6}}_{\pm 0.05}$	$2.39_{\pm 0.1}$	$2.31_{\pm 0.07}$	$2.33_{\pm 0.11}$	$2.29_{\pm 0.06}$

### Appendix E.3. POLYMNIST

**Table A11:** Generation coherence (%) for **POLYMNIST** (higher is better) used for the plots in Figure 4 and Figure A5. We report the average *leave-one-out coherence* as a function of the number of observed modalities. *Joint* refers to random generation of the five modalities simultaneously. Bold and underlined numbers indicate the best and second best scores respectively.

Models	Coherence (%↑)				
	Joint	1	2	3	4
MVAE	$4.0_{\pm 1.49}$	$37.51_{\pm 3.16}$	$48.06_{\pm 3.55}$	$53.19_{\pm 3.37}$	$56.09_{\pm 3.31}$
MMVAE	$25.8_{\pm 1.43}$	$75.15_{\pm 2.54}$	$75.14_{\pm 2.47}$	$75.09_{\pm 2.6}$	$75.09_{\pm 2.58}$
MOPOE	$17.32_{\pm 2.47}$	${\underline{69.37}}_{\pm 1.85}$	${\underline{81.29}}_{\pm 2.34}$	$85.26_{\pm 2.36}$	$86.7_{\pm 2.39}$
NEXUS	$18.24_{\pm 0.89}$	$60.61_{\pm 2.51}$	$72.14_{\pm 2.79}$	$76.81_{\pm 2.75}$	$78.92_{\pm 2.64}$
MVTCAE	$0.21_{\pm 0.05}$	$57.66_{\pm 1.06}$	$78.44_{\pm 1.31}$	${\underline{85.97}}_{\pm 1.43}$	${\underline{88.81}}_{\pm 1.49}$
MMVAE+	$26.28_{\pm 2.19}$	$54.74_{\pm 0.5}$	$54.06_{\pm 0.33}$	$55.2_{\pm 1.32}$	$53.17_{\pm 0.75}$
MMVAE+ (K = 10)	$14.53_{\pm 4.94}$	$58.93_{\pm 6.3}$	$59.42_{\pm 8.8}$	$60.77_{\pm 8.03}$	$58.24_{\pm 7.42}$
MLD IN-PAINT	${\underline{51.65}}_{\pm 1.16}$	$52.85_{\pm 0.23}$	$77.65_{\pm 0.24}$	$85.66_{\pm 0.43}$	$87.29_{\pm 0.29}$
MLD UNI	$48.79_{\pm 0.43}$	$65.12_{\pm 0.7}$	$79.52_{\pm 0.8}$	$82.03_{\pm 1.19}$	$81.86_{\pm 2.09}$
MLD	$56.23_{\pm 0.52}$	$68.58_{\pm 0.72}$	$84.87_{\pm 0.19}$	$88.56_{\pm 0.12}$	$89.43_{\pm 0.27}$

**Table A12:** Generation quality (FID ↓) for **POLYMNIST** (lower is better) used for the plots in Figure 4 and Figure A5. Similar to Table A11, we report the average *leave-one-out* FID as a function of the number of observed modalities. *Joint* refers to random generation of the five modalities simultaneously. Bold and underlined numbers indicate the best and second best scores respectively.

Models	Quality (↓)				
	Joint	1	2	3	4
MVAE	$108.74_{\pm 2.73}$	$108.06_{\pm 2.79}$	$108.05_{\pm 2.73}$	$108.14_{\pm 2.71}$	$108.18_{\pm 2.85}$
MMVAE	$165.74_{\pm 5.4}$	$208.16_{\pm 10.41}$	$207.5_{\pm 10.57}$	$207.35_{\pm 10.59}$	$207.38_{\pm 10.58}$
MOPOE	$113.77_{\pm 1.62}$	$173.87_{\pm 7.34}$	$185.06_{\pm 10.21}$	$191.72_{\pm 11.26}$	$196.17_{\pm 11.66}$
NEXUS	$91.66_{\pm 2.93}$	$207.14_{\pm 7.71}$	$205.54_{\pm 8.6}$	$204.46_{\pm 9.08}$	$202.43_{\pm 9.49}$
MVTCAE	$106.55_{\pm 3.83}$	$78.3_{\pm 2.35}$	$85.55_{\pm 2.51}$	$92.73_{\pm 2.65}$	$99.13_{\pm 2.72}$
MMVAE+	$168.88_{\pm 0.12}$	$165.67_{\pm 0.14}$	$166.5_{\pm 0.18}$	$165.53_{\pm 0.55}$	$165.3_{\pm 0.33}$
MMVAE+ (K = 10)	$156.55_{\pm 3.58}$	$154.42_{\pm 2.73}$	$153.1_{\pm 3.01}$	$153.06_{\pm 2.88}$	$154.9_{\pm 2.9}$
MLD IN-PAINT	$64.78_{\pm 0.33}$	$65.41_{\pm 0.43}$	$65.42_{\pm 0.41}$	$65.52_{\pm 0.46}$	${\underline{65.55}}_{\pm 0.46}$
MLD UNI	$62.42_{\pm 0.62}$	${\underline{63.16}}_{\pm 0.81}$	${\underline{64.09}}_{\pm 1.15}$	${\underline{65.17}}_{\pm 1.46}$	$66.46_{\pm 2.18}$
MLD	${\underline{63.05}}_{\pm 0.26}$	$62.89_{\pm 0.2}$	$62.53_{\pm 0.21}$	$62.22_{\pm 0.39}$	$61.94_{\pm 0.65}$

#### Additional Experiments with the Architecture from [29]

In our experiments on POLYMNIST, we used the same architecture as in [23,27] in order to ensure a fair settings for all the baselines. In [29], the experiments on POLYMNIST were conducted using a different autoencoder architecture based on Resnet instead of a sequence of autoencoder-based convolutional layers. In this section, we investigate the performance of MMVAE+ and our MLD using this architecture. For MMVAE+, we kept the same settings as in [29], including the autoencoder architecture, latent size, and importance sampling K = 10 with doubly reparameterized gradient estimator (DReG). For MLD, we used the same autoencoder architecture with a latent size equal to 160. In Figure A18, can be observed that while the new autoencoder architecture enhances the performance of MMVAE+, the performance our MLD is improved as well. Similar to the previous results, MLD simultaneously achieves the best generative coherence and the best quality.

### Appendix E.4. CUB

**Table A13:** Generation coherence (CLIP-S: higher is better) and quality (FID: ↓ lower is better) for the CUB dataset. **MLD*** denotes the version of our method using a more powerful image autoencoder. Bold numbers indicate the best scores.

Models	Coherence (↑)			Quality (↓)	
	Joint	Image→Caption	Caption→Image	Joint→Image	Caption→Image
MVAE	0.66	0.70	0.64	158.91	158.88
MMVAE	0.66	0.69	0.62	277.8	212.57
MOPOE	0.64	0.68	0.55	279.78	179.04
NEXUS	0.65	0.69	0.59	147.96	262.9
MVTCAE	0.65	0.70	0.65	155.75	168.17
MMVAE+	0.61	0.68	0.65	188.63	247.44
MMVAE+ (K=10)	0.63	0.68	0.62	172.21	178.88
MLD IN-PAINT	0.69	0.69	0.68	69.16	68.33
MLD UNI	0.69	0.69	0.69	64.09	61.92
MLD	0.69	0.69	0.69	63.47	62.62
MLD*	0.70	0.69	0.69	22.19	22.50

### Appendix E.5. CelebAMask-HQ

In this section, we present additional experiments on the CelebAMask-HQ dataset [58].
